# The potential of low‐intensity pulsed ultrasound to apply the long‐term ovary protection from injury induced by 4‐vinylcyclohexene diepoxide through inhibiting granulosa cell apoptosis

**DOI:** 10.1002/btm2.10744

**Published:** 2024-12-17

**Authors:** Juan Deng, Juan Qin, Guolin Song, Chenghai Li, Wentao Tang, Yilin Tang, Xinfang Xiao, Liu Wu, Sicheng He, Yiqing Zhou, Junfen Li, Yan Wang

**Affiliations:** ^1^ State Key Laboratory of Ultrasound in Medicine and Engineering Chongqing Medical University Chongqing China; ^2^ Chongqing Key Laboratory of Biomedical Engineering Chongqing Medical University Chongqing China; ^3^ Department of Obstetrics and Gynecology, Guiyang Maternal and Child Health Care Hospital Guizhou Medical University Guizhou China; ^4^ Department of Emergency The Second Affiliated Hospital of Guizhou University of Traditional Chinese Medicine Guizhou China

**Keywords:** ASK1/JNK, GC apoptosis, LIPUS, ovarian injury, VCD

## Abstract

The potential of low‐intensity pulsed ultrasound (LIPUS) in regulating ovarian function has been demonstrated; however, there is a lack of scientific evidence regarding the long‐term efficacy of LIPUS in treating ovarian injury and understanding its regulatory mechanisms. In this study, 4‐vinylcyclohexene diepoxide (VCD) was used to induce ovarian injury in rats, and LIPUS was applied to target the damaged ovarian tissues. The research aimed to investigate the long‐term protective effect of LIPUS against ovum toxicity induced by VCD and elucidate the associated molecular mechanisms. During the experiment, HE staining was employed for observing the morphology and structure of the ovary, while protein sequencing was utilized for identifying and confirming the molecular mechanism through which LIPUS restores the damaged ovarian structure. The long‐term effectiveness of LIPUS in protecting against ovarian injury was evaluated through ELISA, estrous cycle monitoring, fertility testing, and behavioral analysis. The results indicated that LIPUS effectively restored the structure of damaged ovaries. Both in vivo and in vitro studies revealed that this protective effect may be attributed to LIPUS inhibiting apoptosis of ovarian granulosa cells (GCs) by regulating Daxx‐mediated ASK1/JNK signaling pathway. Subsequent functional tests demonstrated significant improvements in sex hormone secretion and regulation of estrous cycle within 6 cycles following LIPUS treatment. Additionally, there was a notable increase in offspring numbers after mating. Behavioral analysis revealed that LIPUS effectively alleviated menopausal symptoms resulting from ovarian injury including mood fluctuations, cognitive behavior changes, and reduced muscle excitability levels. These findings suggest that beneficial effects of LIPUS may help reduce VCD‐induced ovarian damage with long‐term efficacy.

**Abbreviations**
ELISA: enzyme‐linked immunosorbent assay
GCs: granulosa cells
HE staining: hematoxylin and eosin staining
IHC: immunohistochemistry
KGN: human ovarian granulosa cells
LIPUS: low‐intensity pulsed ultrasound
VCD: 4‐Vinylcyclohexene diepoxide
WB: Western blot analysis

## 1 INTRODUCTION

Ovarian dysfunction is a prevalent cause of female infertility and is associated with genetic, autoimmune, and environmental factors.^1^ These factors can disrupt ovarian metabolism, menstrual cycle regulation, follicle development, and have a significant detrimental impact on female reproductive health.^2^ Previous studies have demonstrated that pathological examinations of persistent ovarian injury caused by various reasons exhibit characteristics such as substantial ovarian atrophy, abnormal follicle development, reduced number of growing follicles, and a marked increase in atretic follicles.^3^ The underlying mechanisms behind these changes are intricate. Animal studies have indicated that senescence and apoptosis of ovarian granulosa cells (GCs) play crucial roles in the decline of ovarian reserve.^4^ Some researchers propose that increased apoptosis of ovarian GCs may accelerate follicular atresia, leading to premature depletion of the original follicle pool. Ultimately, this affects the reproductive lifespan of women in their childbearing years.^5^ Hormone replacement therapy (HRT) has been utilized to compensate for estrogen deficiency in women experiencing ovarian decline and alleviate menopausal symptoms. However, HRT does not address the most pressing need for patients: restoration of their diminished ovarian reserve.^6^ Therefore, it is imperative to pursue a comprehensive solution aimed at alleviating ovarian dysfunction resulting from ovary injury.

As a contemporary physiotherapy, low‐intensity pulsed ultrasound (LIPUS) is a safe and effective non‐invasive therapy that has gradually gained recognition in clinical research.^7^, ^8^ Currently, several studies have demonstrated the significant potential of LIPUS in preserving ovarian function.^9^, ^10^, ^11^, ^12^ In our previous research, we observed a notable increase in the number of primordial and primary follicles in the ovaries of middle‐aged mice exposed to LIPUS water baths. After underwater treatment with LIPUS, there was an improvement in the ovarian structure of mice.^9^ Furthermore, our research team utilized LIPUS to irradiate VCD‐induced ovarian injury in mouse models and preliminarily observed that it could repair ovarian structure and regulate sex hormone secretion.^10^, ^11^ Additionally, there have been reports on the combination of LIPUS with total flavonoids from semen cuscutae alleviating premature ovarian failure by promoting autophagy and inhibiting apoptosis.^12^ With ongoing research progress on exploring the regulation of ovarian function using LIPUS, it provides new possibilities for clinically preventing and treating ovarian injury. However, there is still a lack of studies investigating the long‐term efficacy of LIPUS in treating VCD‐induced ovarian injury and its underlying regulatory mechanism.

The present study aimed to investigate the long‐term protective effect of LIPUS against VCD‐induced ovarian injury and elucidate its potential regulatory mechanisms. LIPUS was employed for the treatment of VCD‐induced ovarian injury in rats. First, we observed the restoration of ovarian structure by LIPUS and proposed that this mechanism may involve the inhibition of ovarian granulosa cell apoptosis through regulation of the Daxx‐mediated ASK1/JNK signaling pathway. Furthermore, this trial continuously monitored and reported on the long‐term effects of LIPUS on ovarioid‐damaged rats over 6 estrous cycles (each rat estrous cycle lasting 4–5 days, equivalent to a human female menstrual cycle occurring every 28–30 days).

## 2 MATERIALS AND METHODS

### 2.1 In vivo test

#### 2.1.1 Animals

A total of 135 SPF‐grade female SD rats, aged 6–8 weeks and weighing 200 ± 10 g, were procured from the Laboratory Animal Center of Chongqing Medical University. The SD rats were housed in a controlled environment with a temperature of 23 ± 5°C, relative humidity of 45% ± 5%, and a light–dark cycle of 12 h each. They were provided with standard food diet and ad libitum access to drinking water. All animal experiments conducted in this study received approval from the Ethics Committee of Chongqing Medical University (Approval number: SCXK 2018–0003). Following 1 week of acclimatization period, the SD rats were utilized for subsequent animal experiments and assigned randomly to different groups using a simple randomization procedure in accordance with the Animal Research: Reporting of In Vivo Experiments (ARRIVE) guidelines version 2.0.

#### 2.1.2 Modeling and LIPUS treatment

After 1 week of adaptive feeding, the 135 rats were randomly divided into three groups: the Control group (*n* = 45), Model group (*n* = 45), and LIPUS group (*n* = 45). Rats in the Model and LIPUS groups received intraperitoneal injections of VCD (Shanghai Aladdin Biochemical Technology Co. LTD, Shanghai, China; 160 mg/kg/day) every day for 15 consecutive days to induce ovarian injury. The estrous cycle was monitored via vaginal smear during the modeling period, and successful model establishment was determined by disrupted cycles. Rats in the Control group received intraperitoneal injections of an equivalent volume of normal saline at consistent times each day.

The LIPUS equipment (Figure S3) utilized in this study was provided by Chongqing Ronghai Engineering Research Center of Ultrasound Medicine Co., Ltd., located in Chongqing, China. Following the successful establishment of the model, depilation was performed on the lower abdomen of the rats and an appropriate amount of ultrasonic coupling agent was applied for LIPUS irradiation. The rats in the LIPUS group received daily ultrasound irradiation for 20 min over a period of 15 days (with an acoustic intensity of 200 mW cm^−2^, focal plane distance set at 10.0 mm which corresponds to a positive acoustic pressure of 0.30 MPa, negative acoustic pressure of −0.31 MPa, and peak‐to‐peak acoustic pressure of 0.61 MPa (The hydrophone detection diagram is shown in Figure S4); frequency set at 0.36 MHz; repetition frequency at 1 kHz; duty cycle at 20%; utilizing a single‐element piezoelectric planar transducer with a diameter measuring 25 mm). Additionally, the Model group underwent sham treatment simultaneously (using an ultrasonic probe without energy output).

#### 2.1.3 Estrous cycle detection

The estrous cycle of the rats was monitored by continuously collecting vaginal smears throughout the experiment to assess ovarian function. Exfoliated vaginal cells were gently collected using cotton swabs and evenly spread onto slides. Subsequently, the slides were fixed with a 95% ethanol solution (Chongqing Chuandong Chemical Co., Ltd., Chongqing China) and stained with 0.23% alkaline methylene blue (Beijing Solar Biotechnology Co., Ltd., Beijing China). Finally, an optical microscope (BX51, Olympus, Tokyo, Japan) was employed to observe and record the estrous cycle of the rats. Disturbances in the estrous cycle serve as indicators of impaired ovarian function and reduced reproductive capacity.^13^

#### 2.1.4 Histopathological examination (H&E)

After LIPUS treatment, rats from each group were euthanized, and their ovaries, hearts, livers, spleens, lungs, and kidneys were excised and fixed in 4% paraformaldehyde for 48 h. The tissues were subsequently embedded in paraffin, sectioned, stained with H&E dye, and examined under an optical microscope. Morphological and structural changes of various organs were assessed along with the quantification of primordial follicles, primary follicles, secondary follicles, mature follicles, and atretic follicles in the ovarian tissue. Statistical analysis was performed.^14^, ^15^

#### 2.1.5 Enzyme‐linked immunosorbent assay (ELISA)

After LIPUS treatment, serum samples were collected from rats in each group. The levels of sex hormones (FSH, LH, E2, and AMH) in rat serum were quantified using an ELISA kit (Jiangsu Jingmei Biological Technology Co., Ltd., Jiangsu, China) that had been pre‐coated with specific antibodies. The entire experimental procedure was conducted strictly following the manufacturer's instructions.

#### 2.1.6 TdT‐mediated dUTP nick end labeling (TUNEL)

The paraffin sections of ovarian tissue were dewaxed in xylene, dehydrated in graded alcohol, and subsequently washed with PBS. Following this, the sections were incubated with protease K at room temperature for a duration of 20 min. Subsequently, the TUNEL kit (Shanghai Biyuntian Biotechnology Co., Ltd., Shanghai, China) was employed to detect apoptosis of ovarian granulosa cells (GCs), following the manufacturer's instructions. The staining results were observed using a laser scanning confocal microscope (LEICA TCS SP8, Germany), with apoptotic cells in the ovary appearing green.

#### 2.1.7 iTRAQ data analysis and bioinformatic analysis

The rat ovary tissue in each group was subjected to protein TMT quantification by Wuhan Huada Medical Inspection Co., Ltd. using IQuant software. Following protein extraction and identification, TMT quantitative analysis was performed, with proteins exhibiting significant differences in expression selected under the condition of Foldchange >1.2 and *Q*‐value <0.05. Finally, cluster analysis and KEGG enrichment analysis were utilized to identify key pathways involved in LIPUS treatment of POI.

#### 2.1.8 Immunohistochemistry (IHC)

The ovarian tissues of rats in each group were subjected to dewaxing and antigen retrieval. Subsequently, the sections were incubated with primary antibodies (Fas, Daxx, ASK1, JNK, Bcl‐2, Bax, Cytc, Apaf‐1, Caspase 3, Caspase 9, and PARP) (Affinity Biosciences LTD., OH, USA) at 37°C for 1 h. Following that, the biotinylated secondary antibody anti‐rabbit IgG (Fuzhou Maixin Biotechnology Development Co. LTD., Fuzhou, China) was added and incubated for 20 min at room temperature.^16^ Finally, DAB and hematoxylin staining was performed on each section and five different areas were randomly selected for image capture under an optical microscope. All images were quantitatively analyzed for protein expression using ImageJ software (NIH, Bethesda, MD, USA).

#### 2.1.9 Western blot

The ovarian tissue was subjected to protein extraction using RIPA buffer, and the protein concentration was determined utilizing a BCA protein detection kit (Beyotime, Beijing, China). Subsequently, the protein samples were separated on SDS‐PAGE, transferred onto a PVDF membrane, and blocked with 5% nonfat milk. Following this step, the membrane was incubated overnight at 4°C with primary antibodies specifically targeting Fas, Daxx, ASK1, JNK, Bcl‐2, Bax, Cytc, Apaf‐1, Caspase 3, Caspase 9, PARP, and β‐actin (Affinity Biosciences Ltd., OH, USA). Afterward, the membrane underwent incubation with the secondary antibody at room temperature for 1 h. Finally, the Western blot results were evaluated using the GenGnome imaging system (Syngene, Cambridge, UK), and quantitative analysis of different proteins' expression levels was performed using ImageJ software.

#### 2.1.10 Quantitative real‐time PCR (qRT‐PCR)

Total RNA was extracted from ovarian tissue using an RNA rapid extraction kit (Nanjing Vazyme Biology Co., Ltd., Nanjing, China). The concentration of RNA was determined using an enzyme labeling instrument. Reverse transcription of the extracted RNA was performed according to the instructions of the PrimeScript™ RT reagent Kit (TaKaRa, Tokyo, Japan) to obtain 20 μL cDNA. qPCR analysis was conducted on a Real‐time fluorescence quantitative PCR instrument (Bio‐Rad, USA) using ChamQ Universal SYBR qPCR Master Mix (Nanjing Vazyme Biology Co., Ltd., Nanjing, China) and specific primers (Shanghai Shenggong Biological Engineering Co., Ltd., Shanghai, China) for Fas, Daxx, ASK1, JNK, Bcl‐2, Bax, Cytc, Apaf‐1, Caspase 3, Caspase 9, and PARP mRNA expression. The specific primers used are listed in Table S1.

#### 2.1.11 Open field test

The rats were placed in a square open field box measuring 100 cm × 100 cm × 40 cm, and their activity was recorded for a duration of 5 min using a camera. The data collected from each group of rats were analyzed utilizing SMART 25.21 software. The central area was defined as the region occupying the middle quarter of the bottom surface of the open field box, while the remaining portion was considered as the peripheral area. Parameters such as total distance covered during activity, total distance covered within the central area, total time spent in the central area, and average speed were measured and recorded for each group of rats.

#### 2.1.12 Grip test

The front paws of the rats in each group were positioned on the grip tester, and a gentle traction was applied to the tail of the experimental rats until they reached their maximum grip strength. The highest value achieved was recorded. This measurement process was repeated five times for each rat to obtain an average value, which serves as an indicator of the muscle strength exhibited by the experimental rat.

#### 2.1.13 Pregnancy rate detection

The female rats from each group were paired with sexually mature male rats at a 1:1 ratio, and the fertility was continuously observed and documented.

### 2.2 In vitro test

#### 2.2.1 KGN cell culture, modeling, and LIPUS treatment

In vitro experiments were performed using KGN cells (Shanghai Fuheng Biotechnology Co., Ltd., China), which were cultured in DMEM medium (Thermo, USA) supplemented with 10% fetal bovine serum (Gibco, USA) and 1% penicillin–streptomycin at 37°C with 5% CO_2_ in a humidified environment. The cells were seeded at a density of 5 × 10^5^ cells/well in a 6‐well plate and incubated for 24 hours before exposure to varying concentrations of VCD (0–4 mmol/L) for different time periods. Cell viability was measured by absorbance values (OD) at 490 nm using an enzyme marker, and the experiment was repeated three times. After selecting the concentration closest to IC50, the experiment was divided into four groups: Control group, Model group, LIPUS group, and Ac‐DEVD‐CHO group. The LIPUS equipment (Figure S5) utilized in this study was provided by Chongqing Ronghai Engineering Research Center of Ultrasound Medicine Co., Ltd., located in Chongqing, China. Following successful modeling, the LIPUS group received LIPUS treatment for 3 days with acoustic intensity of 30 mW cm^−2^, focal plane distance of 1.0 mm corresponding to positive acoustic pressure of 0.20 MPa and negative acoustic pressure of −0.25 MPa resulting in peak‐to‐peak acoustic pressure of 0.45 MPa (The hydrophone detection diagram is shown in Figure S6); frequency was set at 0.36 MHz; single‐element piezoelectric planar transducer had diameter of 25 mm; repetition frequency was set at 1 kHz and duty cycle was 20%. The KGN cells in Ac‐DEVD‐CHO group were treated with Caspase3 inhibition.

#### 2.2.2 CCK‐8 cell viability assay

The cell proliferation of KGN was assessed using the CCK8 kit (Beijing Solaibao Biotechnology Co., LTD). Cells were seeded at a density of 5 × 10^3^ cells per well in a 96‐well plate and incubated for 24 h. On the subsequent day, each well received 10 μL of CCK8 solution and was further incubated for 3 h. Absorbance at 450 nm was measured using an enzyme marker, and the resulting value was calculated and analyzed following the manufacturer's instructions.

#### 2.2.3 TUNEL

The KGN cells were subjected to a single wash with PBS buffer, followed by fixation in 4% paraformaldehyde for 30 min. Subsequently, they were incubated in PBST (PBS containing 0.3% Triton X‐100) at room temperature for 5 min. Detection of apoptosis in ovarian granulosa cells was performed using the TUNEL kit according to the provided instructions. Finally, the cells were visualized under a fluorescence confocal microscope.

#### 2.2.4 Flow cytometry analysis

The floating cells in the medium were collected and transferred into centrifuge tubes, while the adherent KGN cells in 6‐well plates were dissociated into single cells using 0.25% trypsin (Hyclone, USA). Subsequently, the cells were pelleted by centrifugation at 1000 rpm for 5 min with PBS buffer (Hyclone, USA), and the supernatant was discarded. This process was repeated twice. Finally, a suspension of 1 × 10^6^ cells in 500 μL PBS was prepared and transferred to 1.5 mL EP tubes for machine detection.

#### 2.2.5 Assay of mitochondrial membrane potential

The working solution for TMRE staining was prepared according to the instructions provided by Shanghai Biyuntian Biotechnology Co., LTD, which is a mitochondrial membrane potential detection kit. One milliliter of TMRE was added to each well and incubated at 37°C for 30 min. The cells were then washed twice with PBS and subsequently incubated with DAPI staining solution at room temperature for 15 min. Finally, the samples were observed under a fluorescence confocal microscope, where the intensity of red fluorescence indicated the level of mitochondrial membrane potential. Additionally, quantitative analysis was also conducted using flow cytometry.

#### 2.2.6 Western blot

The KGN cells were subjected to protein extraction using a RIPA buffer solution, followed by determination of protein concentration using a BCA protein detection kit. All subsequent procedures were performed in accordance with in vivo experiments.

#### 2.2.7 Statistical analysis

The experimental data were analyzed using GraphPad Prism Version 8 (GraphPad Software Inc., San Diego, CA). The results are presented as mean ± SD, and differences between groups were assessed using one‐way analysis of variance. Statistical significance was defined as *p* < 0.05.

## 3 RESULTS

### 3.1 The LIPUS treatment effectively restored the morphological structure of the ovary and facilitated the normal development of follicles

**FIGURE 1:**
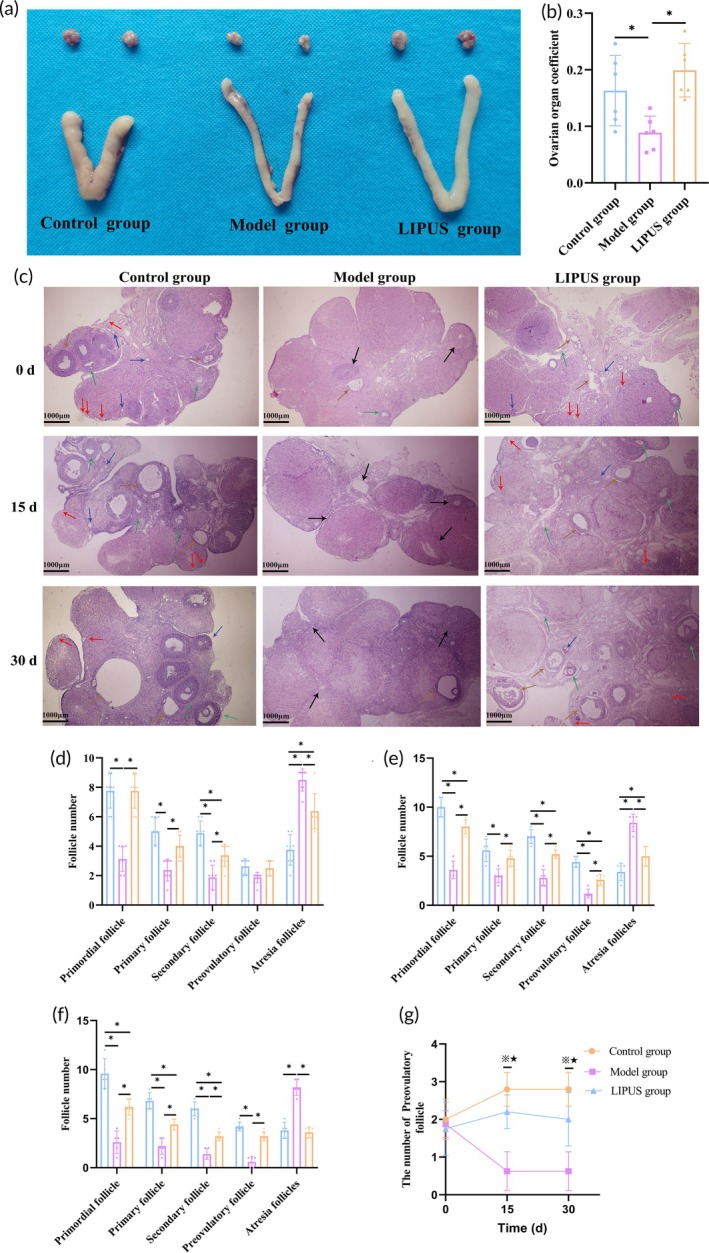
The findings suggest that LIPUS has the potential to restore the damaged ovarian structure caused by chemical drugs and promote normal follicle development. (a) Morphological comparison of reproductive organs was conducted in each group. (b) The coefficient of ovarian tissue mass was compared before and after LIPUS treatment. (c–f) Ovarian histopathological analysis (40×) was performed, and the counts of primordial, primary, secondary, mature, and atretic follicles were evaluated in the ovaries of rats in each group at day 0 d, 15 d, and 30 d after LIPUS treatment (Red arrow: Primordial follicles; Blue arrow: Primary follicle; Green arrow: Secondary follicles; Yellow arrow: Preovulatory follicle; Black arrow: Atresia follicle). (g) Changes in the number of mature follicles were observed in rats during different time periods. *p < 0.05, ※*p* < 0.05, ★*p* < 0.05.

The morphology of reproductive organs was significantly restored after treatment with LIPUS, as depicted in Figure 1a,b. Pathological sections of ovarian tissue revealed a significant restoration of the ovarian structure in rats treated with LIPUS (Figure 1c–f). On day 0 following LIPUS treatment, there was a significant increase in the number of primordial follicles, primary follicles, and secondary follicles in the Model group, accompanied by a notable decrease in atretic follicles (*p* < 0.05). However, no significant difference was observed in the number of mature follicles (*p* > 0.05). Nevertheless, on the 15th and 30th days, pathological sections demonstrated a significant decrease in the number of mature follicles within the Model group (*p* < 0.05) (Figure 1g). These findings suggest that LIPUS has reparative effects on chemically damaged ovarian structures and promotes normal follicle development.

### 3.2 The administration of LIPUS effectively suppressed the apoptosis of ovarian granulosa cells in rats with VCD‐induced ovarian injury

**FIGURE 2:**
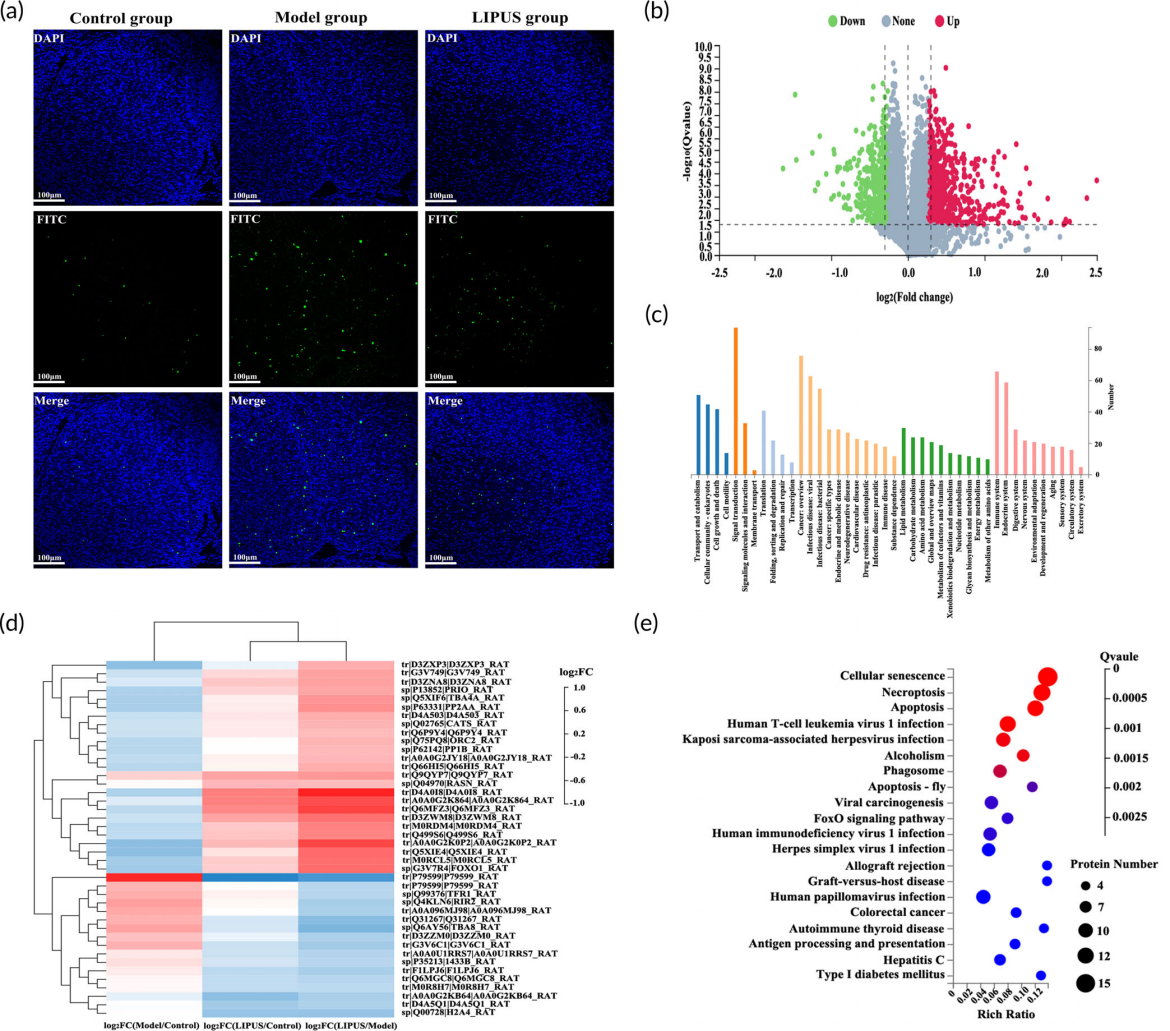
The administration of LIPUS effectively suppressed the apoptosis of ovarian granulosa cells (GCs) in rats with VCD‐induced ovarian injury. (a) TUNEL staining was performed to observe the apoptosis of ovarian GCs in each group, where blue represents the nucleus of GCs and green fluorescence indicates apoptotic cells (400× magnification). (b) Volcano plot illustrating changes in differential proteins within the ovarian tissue of rats induced by LIPUS treatment. (c) Functional annotation through KEGG enrichment analysis for differential protein identification. (d) Cluster heatmaps displaying 44 distinct proteins associated with cell growth and death. (e) Bubble map depicting 44 different proteins related to cell growth and death after KEGG enrichment analysis.

To investigate the impact of LIPUS on ovarian granulosa cells (GCs), we initially conducted a TUNEL assay. The findings demonstrated a reduction in apoptosis of ovarian GCs in rats subjected to LIPUS treatment (Figure 2a). Subsequently, proteomic analysis was employed to elucidate the underlying mechanism. Utilizing iTRAQ quantitative proteomics, we identified 72,210 peptides and 8707 proteins at a false discovery rate (FDR) filtering standard of 1%. The volcano plot unveiled 1141 differentially expressed proteins between the Model group and the LIPUS group, with 678 proteins up‐regulated and 463 proteins down‐regulated (Figure 2b).

To refine the target range and enhance specificity, we conducted KEGG functional annotation analysis on the differential proteins associated with cell growth and death (42 in total), as depicted in Figure 2c. The differential cluster heat map revealed that expression trends of these proteins were opposite between the Model group and LIPUS group, while they remained consistent between the LIPUS group and Control group (Figure 2d). Notably, KEGG analysis indicated significant enrichment of these differential proteins on the apoptosis signaling pathway, including a total of 11 closely related proteins (Figure 2e). These findings suggest that LIPUS can effectively suppress ovarian GC apoptosis in model rats.

### 3.3 The Daxx‐mediated ASK1/JNK signaling pathway was significantly inhibited by LIPUS

The proteomic analysis revealed a prominent enrichment of the apoptotic signaling pathway among the differentially expressed proteins between pre‐ and post‐LIPUS treatment. Notably, Daxx exhibited the most significant difference in expression. This finding prompted further investigation into the role of Daxx in mediating the ASK1/JNK signaling pathway. Protein and mRNA expression levels of these apoptosis‐related factors were quantitatively assessed using immunohistochemical staining, Western blot, and qPCR (Figures S1 and S8, 3a–c). The results demonstrated that compared to the Model group, ovarian tissue in the LIPUS group showed significantly decreased expressions of Fas, Daxx, ASK1, JNK, Bax, Cytc, Caspase9, Caspase3, PARP, and other pro‐apoptotic factors. Conversely, the anti‐apoptosis factor Bcl‐2 was significantly increased (*p* < 0.05). These findings suggest that LIPUS may mitigate ovarian damage by inhibiting the Daxx‐mediated ASK1/JNK signaling pathways.

**FIGURE 3:**
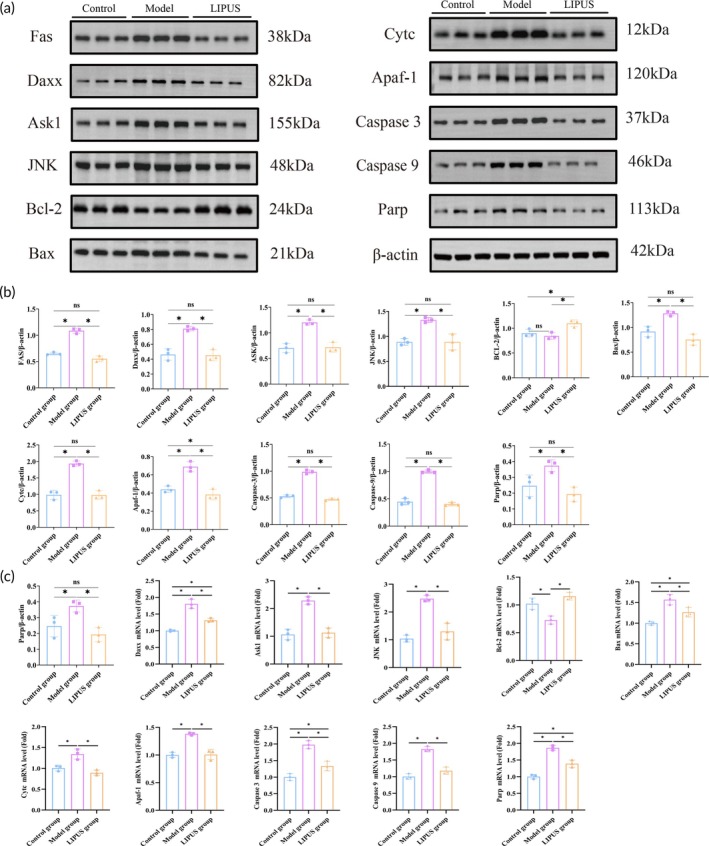
The LIPUS treatment exerts an inhibitory effect on apoptosis of GCs by modulating the Daxx‐mediated ASK1/JNK signaling pathway. (a, b) Representative Western blot image depicting the proteins involved in the Daxx‐mediated ASK1/JNK signaling pathway, along with quantitative analysis of these proteins. (c) mRNA expression levels of relevant proteins were assessed. *p < 0.05.

### 3.4 The administration of LIPUS effectively suppressed VCD‐induced mitochondrial damage and attenuated apoptosis in KGN cells

**FIGURE 4:**
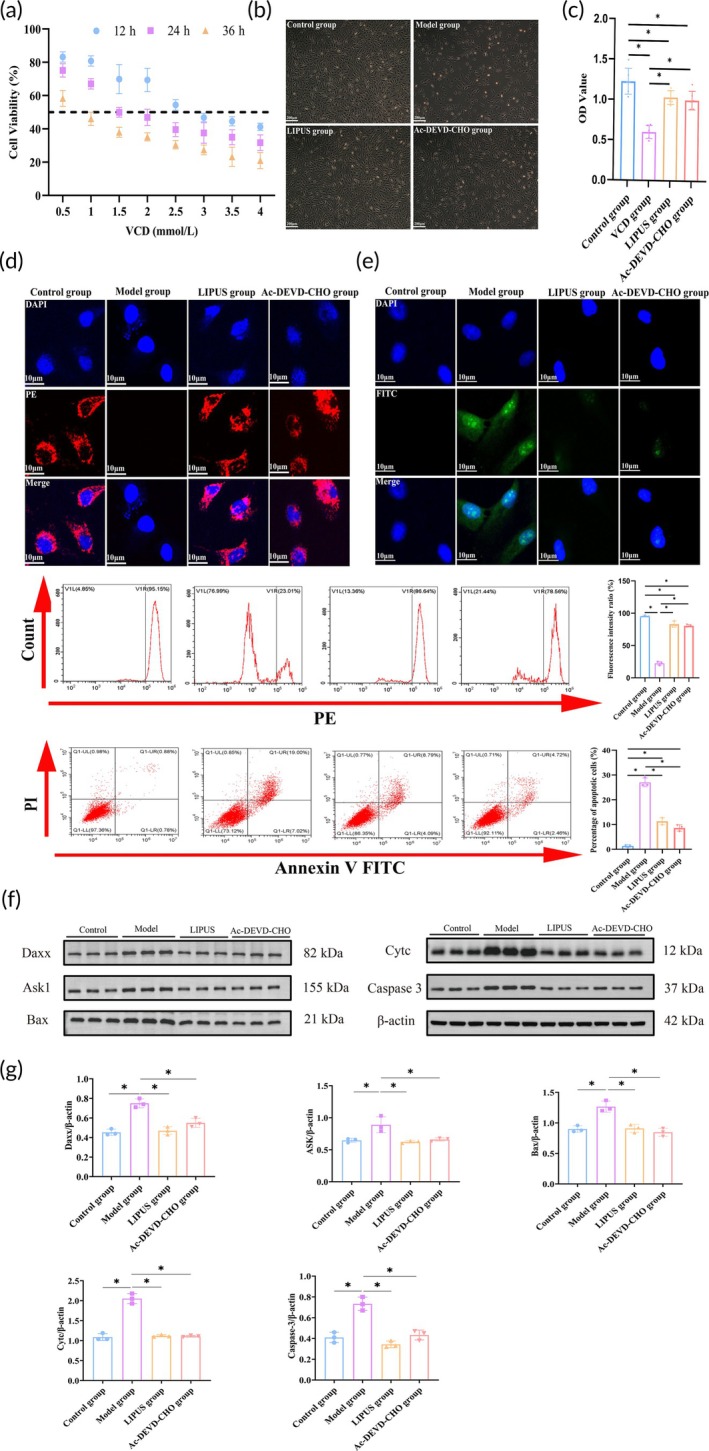
LIPUS effectively suppressed VCD‐induced apoptosis of KGN cells. (a) The proliferation of KGN cells was assessed under various concentrations of VCD and different treatment durations. (b) Cell growth in each group was evaluated after 3 days of LIPUS intervention. (c) CCK8 assay was performed to determine the cellular activity in each group. (d) TMRE staining revealed the mitochondrial membrane potential levels, which were quantitatively analyzed using flow cytometry at a magnification of 1200×. (e) TUNEL staining was used to observe apoptosis, and quantitative analysis was conducted by flow cytometry at a magnification of 1200×. (f) Western blotting detected the expression levels of Daxx, ASK1, Bax, Cytc, and Caspase 3 in cells from each group. (g) Quantitative analysis demonstrated the changes in Daxx, ASK1, Bax, Cytc, and Caspase 3 expression levels among groups. *p < 0.05.

To further validate the results of the animal experiments, we conducted in vitro experiments. Figure 4a illustrates the proliferation of KGN cells following exposure to varying concentrations of VCD (0, 0.5, 1, 1.5, 2, 2.5, 3, 3.5, and 4 mmol/L). After careful calculation and considering IC50 values as a reference point, a reaction time of 24 hours and a concentration of approximately IC50 (1.5 mmol/L) were selected as optimal conditions for subsequent experiments.

We initially assessed cell proliferation in each group after 3 days of LIPUS treatment and observed significantly higher rates in both the LIPUS and Ac‐DEVD‐CHO groups compared to the Model group (Figure 4b). Subsequently, we compared KGN activity among these treated groups (Figure 4c). The results demonstrated that LIPUS treatment led to a significant increase in KGN activity (*p* < 0.05), with levels comparable to those observed in the Ac‐DEVD‐CHO group. Conversely, the Model group exhibited significantly reduced cell activity when compared to the Control group (*p* < 0.05). These findings suggest that LIPUS has potential for enhancing KGN activity.

As depicted in Figure 4d, the level of mitochondrial membrane potential was assessed in each group through TMRE staining. The results revealed a significant increase in red fluorescence intensity within the mitochondria of KGN cells treated with LIPUS and Ac‐DEVD‐CHO, while the red fluorescence almost disappeared in the Model group. Furthermore, flow cytometry was employed to quantitatively analyze the mitochondrial membrane potential of KGN cells across all groups. The findings demonstrated a significant elevation in mitochondrial membrane potential levels in the LIPUS group compared to the Model group (*p* < 0.05). Conversely, the Model group exhibited a substantial reduction in membrane potential when compared to the Control group (*p* < 0.05). Then, we observed the apoptosis of KGN in each group by TUNEL staining and flow cytometry (Figure 4e). The results of TUNEL staining showed that the green fluorescence of KGN in the LIPUS group and Ac‐DEVD‐CHO group was significantly lower than that in the Model group. The results of flow cytometry showed that the apoptosis of KGN treated with LIPUS and Ac‐DEVD‐CHO decreased significantly (*p* < 0.05), while that of the Model group increased significantly compared with the Control group (*p* < 0.05). These results confirm that the effect of LIPUS is comparable to that of Ac‐DEVD‐CHO, significantly inhibiting KGN apoptosis. Additionally, Western blotting was performed to detect the expression of related proteins in the Daxx‐mediated ASK1/JNK apoptosis signaling pathway (Figures 4f,g and S8). The findings revealed a significant decrease in the expression of pro‐apoptotic factors including Daxx, ASK1, Bax, Cytc, Caspase 3, and PARP in both the LIPUS group and Ac‐DEVD‐CHO group (*p* < 0.05), while an increase in anti‐apoptotic factor Bcl‐2 was observed. Conversely, compared to the Control group (*p* < 0.05), these pro‐apoptotic factors were significantly increased and anti‐apoptotic factors were significantly decreased in the Model group.

These findings suggest that LIPUS can mitigate the loss of mitochondrial membrane potential and suppress KGN apoptosis by modulating the Daxx‐mediated ASK1/JNK mitochondrial apoptosis signaling pathway, with a similar effect to Ac‐DEVD‐CHO.

### 3.5 The application of LIPUS has demonstrated significant efficacy in restoring ovarian function in rats with VCD‐induced ovarian injury, and this therapeutic effect can be sustained for up to 6 estrous cycles

**FIGURE 5:**
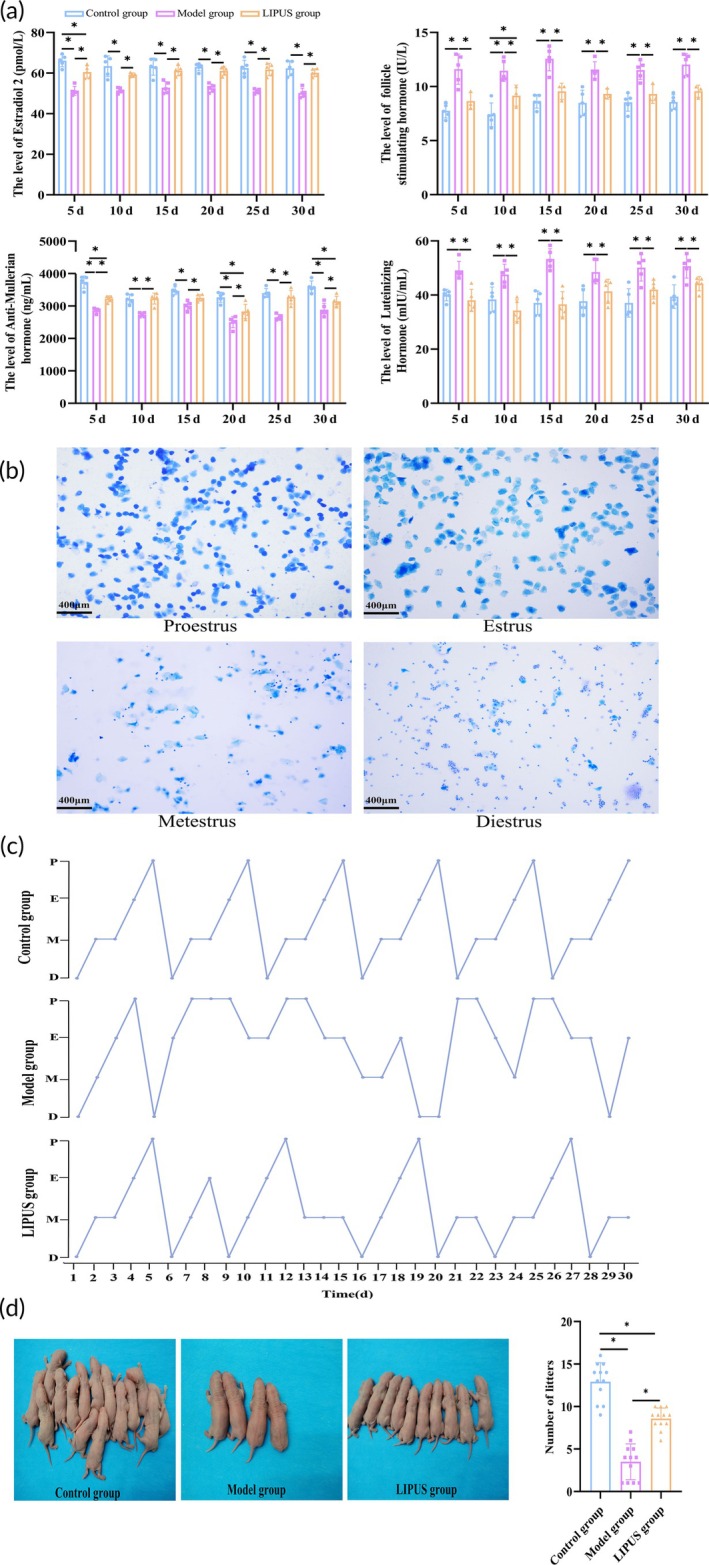
The therapeutic effects of LIPUS on VCD‐induced ovarian injury in rats were observed for up to 6 estrous cycles, as evidenced by the restoration and maintenance of ovarian function. (a) Serum levels of E2, FSH, AMH, and LH were measured at 5, 10, 15, 20, 25, and 30 days post LIPUS treatment in each group. (b) Morphological images of vaginal exfoliation cells during proestrus, estrus, metestrus, and diestrus stages were examined in rats. (c) The dynamic changes in the estrous cycle within a period of 30 days after LIPUS treatment were monitored in all groups. (d) The number of offspring resulting from mating was assessed for each group of rats. *p < 0.05.

The LIPUS‐treated rats demonstrated a tendency toward restoration of normal sex hormone production within 30 days, as depicted in Figure 5a,b, without any observed rebound effect (Figure 5c). Moreover, we conducted dynamic monitoring of the estrus cycle in 30‐day‐old rats and found that the intersection between the estrus cycles of rats in the LIPUS group and those in the Model group occurred within 30 days, indicating a significantly higher level of normalization (Figure 5d,e). In terms of mating experiments, it was observed that the number of pups significantly increased in the LIPUS group compared to the Control group (*p* < 0.05), while there was a significant decrease in pup numbers in the Model group (*p* < 0.05). Additionally, we performed a safety evaluation on LIPUS and found no significant injuries to surrounding organs (Figure S2). These results further confirm that LIPUS can effectively restore ovarian function impaired by VCD.

### 3.6 The symptoms of menopause caused by ovarian decline can be alleviated by LIPUS

**FIGURE 6:**
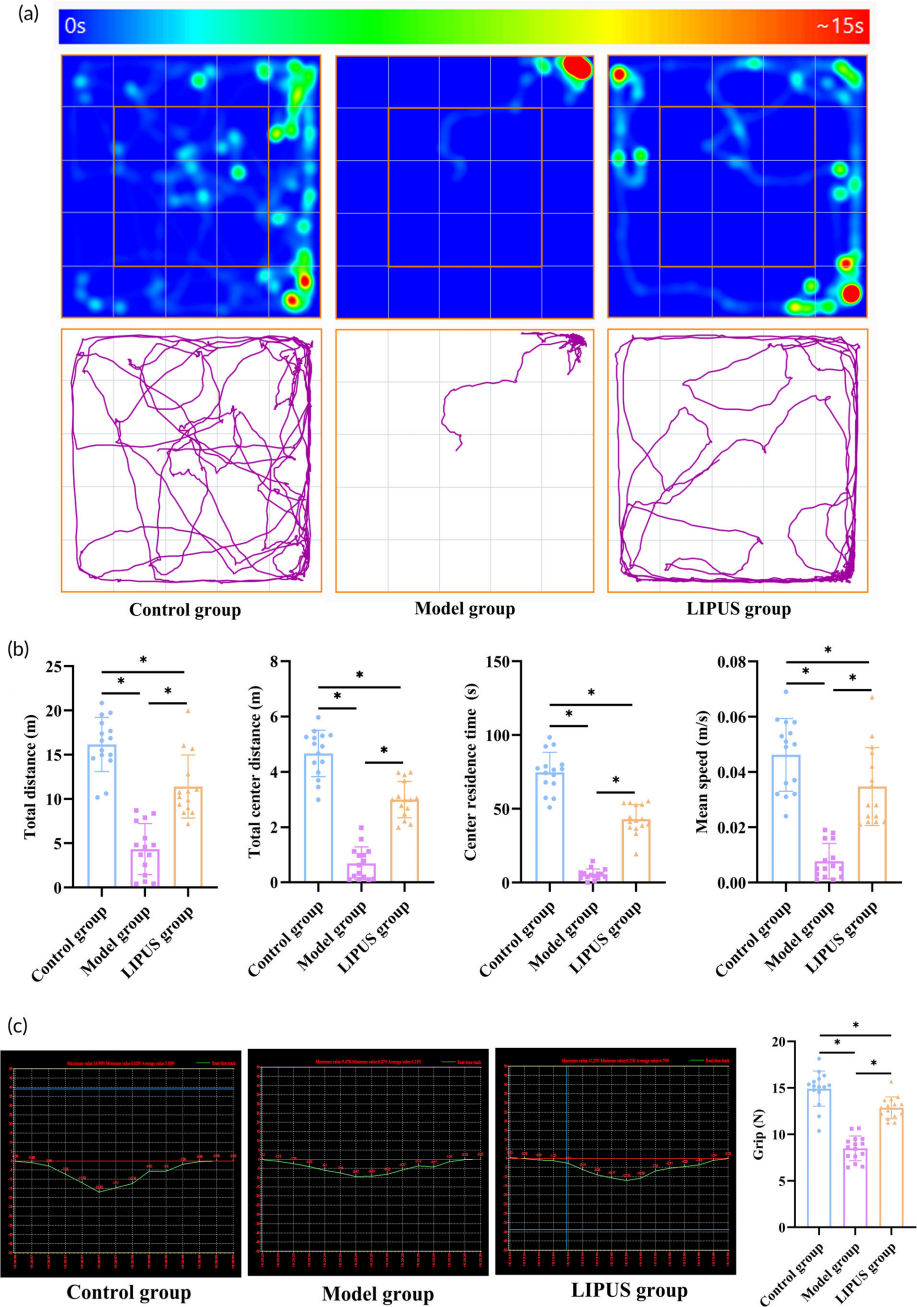
The use of LIPUS has been shown to alleviate menopausal symptoms resulting from ovarian decline. (a) An activity heat map and activity track were generated for each group of rats during the open field test. (b) Measurements such as total moving distance, residence time, total moving distance, and average velocity were taken to assess the levels of activity, behavioral cognition, and depression in rats during the open field test. (c) Representative images and quantitative analysis were conducted to evaluate the gripping power of rats in each group. *p < 0.05.

The motion heat map and motion track map of each group of rats in the open field test are depicted in Figure 6a. Statistical analysis of the experimental data revealed that the LIPUS group exhibited significantly higher total exercise distance, stay time in the central area, total exercise distance in the central area, and average speed compared to the Model group (*p* < 0.05). Conversely, these indices were significantly decreased in the Model group when compared to the Control group (*p* < 0.05) (Figure 6b). The grip test results demonstrated a significant increase in maximum grip strength among rats in the LIPUS group as opposed to those in the Model group, while it was significantly lower for rats in the Model group than for those in the Control group (*p* < 0.05) (Figure 6c). These findings suggest that LIPUS may alleviate menopausal symptoms such as depression, behavioral cognitive impairment, and reduced muscle excitability caused by ovarian injury.

## 4 DISCUSSION

The ovary, as the central reproductive organ in women, plays a crucial role in maintaining normal reproductive function and endocrine system stability. Any form of ovarian damage can have a significant impact on a woman's life.^17^ Although numerous treatments for disorders associated with ovarian dysfunction have been proposed, none of them are universally regarded as the most effective in simultaneously restoring damaged ovarian function and being universally applicable.^18^, ^19^ Fortunately, progressive non‐invasive physical therapies like LIPUS are gaining attention from clinical researchers. Previous studies have demonstrated the potential of LIPUS in regulating ovarian function.^10^, ^11^, ^12^ This study further validates the protective effect of LIPUS on VCD‐induced structural damage to the ovaries and elucidates that its potential mechanism involves reducing mitochondrial damage through regulation of the Daxx‐mediated ASK1/JNK signaling pathway, thereby inhibiting apoptosis in ovarian granulosa cells (GCs). Additionally, this study observes long‐term efficacy of LIPUS by effectively restoring ovarian reserve function and alleviating menopausal symptoms resulting from ovarian injury; this beneficial effect lasts for at least 6 estrous cycles.

The VCD compound is a novel substance with ovarian toxicity that specifically targets small ovarian follicles for degradation, resulting in a reduction of the ovarian reserve.^20^ In this study, we utilized VCD induction to establish a rat model of chemically induced ovarian injury and observed that exposure to VCD enhanced apoptosis in developing follicles and granulosa cells, leading to damage in the female rats' reproductive system. These findings are consistent with previously reported results.^21^, ^22^, ^23^ Furthermore, targeted LIPUS irradiation was applied to the damaged sites within the ovaries of rats and changes in their morphology and structure were monitored. This initially confirmed the protective effect of LIPUS on VCD‐induced ovarian injury in the rat model as it restored their morphology and promoted normal development of ovarian follicles. This protective effect aligns with most relevant studies.^24^, ^25^

The status of granulosa cells (GCs) plays a crucial role in follicular development, and the main mechanisms for restoring ovarian function involve regulating GC apoptosis and repairing primordial follicular reserve.^26^ Proteomic analysis in this study revealed that treatment in the LIPUS group significantly enriched 42 proteins related to cell growth and death in the apoptotic signaling pathway. Apoptosis is a physiological programmed cell death mechanism involving various signals and regulatory pathways.^27^ However, most studies on apoptosis have focused on the Bcl‐2 and Bax families. Daxx, as a multifunctional protein, has been reported to act as a transcriptional co‐repressor in the nuclear compartment with HDAC1, DNA methyltransferase 1 (DMNT1), and α‐thalassemia/mental retardation syndrome X‐linked (ATRX).^28^ Besides its nuclear role, studies have found that Daxx is involved in the caspase‐independent pathway of apoptosis by activating apoptosis signal‐regulated kinase 1 and c‐JunNH2 terminal kinase 4 in the cytoplasm.^29^, ^30^ Nevertheless, there are limited studies on Daxx's involvement in GCs. The expression of Daxx was found to be significantly upregulated in human ovarian cancer tissues, while weakly expressed in normal ovarian tissues.^28^ Surprisingly, our study revealed a significant difference in the expression of Daxx between the ovarian tissues of rats in the LIPUS group and Model group. Previous studies by Liu et al. have demonstrated that Daxx can induce cell apoptosis through activation of the ASK1/JNK signaling pathway, resulting in cytc release.^31^ Consistent with these findings, we observed a significant increase in the expression levels of pro‐apoptotic factors associated with this pathway both in vitro and in vivo trials conducted on the Model group, accompanied by a decrease in anti‐apoptotic factors. However, treatment with LIPUS reversed the expression levels of these factors within ovarian tissue. Based on our findings, we propose that targeting Daxx could be a potential approach for LIPUS‐mediated regulation of apoptosis in ovarian granulosa cells (GCs) following VCD‐induced ovarian injury.

We further assessed the mitochondrial membrane potential of KGN cells in each experimental group through TMRE staining in an in vitro study. Mitochondria are highly abundant in GCs and play a critical role in cell proliferation, energy generation, and apoptosis regulation. Previous studies have indicated that during ovarian function decline, mitochondrial function becomes compromised, resulting in inadequate energy supply, increased free radical production, diminished mitochondrial membrane potential, and release of intracellular calcium ions that activate the intracellular apoptosis pathway leading to GC apoptosis.^32^, ^33^ Our findings align with this concept as apoptotic KGN cells induced by VCD exhibited nearly abolished mitochondrial membrane potential while treatment with LIPUS significantly elevated the level of mitochondrial membrane potential in KGN cells. Based on these collective observations, we propose that LIPUS can mitigate excessive mitochondrial damage by modulating the Daxx‐mediated ASK1/JNK signaling pathway. This intervention may contribute to preserving normal levels of mitochondrial membrane potential, inhibiting Cytc release, and ultimately reducing GC apoptosis.

A common limitation of our previous studies and other related research is the absence of long‐term efficacy observation.^34^ Unlike the oophorectomy (OVX) model, which exhibits sudden loss of ovarian function, the VCD‐induced ovarian injury model simulates human ovarian decline in a progressive manner, consistent with clinical characteristics of POI patients.^35^ This unique advantage necessitates an extended observation period to further investigate the effectiveness of LIPUS. Building upon our understanding of LIPUS's effectiveness and regulatory mechanism in restoring ovarian structure in rats with ovarian injury, this study extends its focus to observe the long‐term effects on regulating ovarian reserve function. The results demonstrate that hormone secretion levels, estrous cycle patterns, and fertility in rats were significantly restored within 6 estrous cycles following LIPUS treatment. Furthermore, menopausal symptoms resulting from declining ovarian function were notably alleviated. This long‐term observation addresses limitations present in previous studies while acknowledging certain limitations within this study itself. Additionally, it highlights the need for future research to explore potential effects of variations in LIPUS parameters on ovarian regulation.

In conclusion, this study presents novel evidence suggesting that LIPUS exerts a prolonged protective effect against VCD‐induced ovarian injury by modulating the Daxx‐mediated ASK1/JNK signaling pathway, suppressing mitochondrial damage, enhancing the growth and metabolic activity of ovarian GCs, thereby facilitating the restoration of ovary structure and function in rats. The identification of its sustained efficacy and regulatory mechanism offers a promising avenue for clinical management of female ovarian injury.

## AUTHOR CONTRIBUTIONS

J.D. and Y.W. designed the experiment. J.Q., G.S., C.L., and W.T. are responsible for the bioinformatics analysis. Y.T. conducted animal experiments. X.X. and L.W. conducted molecular experiments. S.H., Y.Z., and J.L. are responsible for supervising animal and molecular experiments. Y.W. supervised the entire experiment. All the authors contributed to writing and reviewing the manuscript.

## FUNDING INFORMATION

This work was supported by the Basic Research and Frontier Exploration Project of Yuzhong District, Chongqing (20210133), Natural Science Foundation project of Chongqing Science and Technology Bureau (cstc2021jcyj‐msxmX0487), Chongqing Graduate Research Innovation Project (CYS22373), Program for Youth Innovation in Future Medicine, Chongqing Medical University (W0155), and National Natural Science Foundation of China (12004059).

## CONFLICT OF INTEREST STATEMENT

The authors declare that they have no competing interests.

## Supporting information


Data S1.

## Data Availability

All data generated or analyzed during this study are included in this published article.

## References

[1] Wang J , Liu W , Yu D , Yang Z , Li S , Sun X . Research progress on the treatment of premature ovarian failure using mesenchymal stem cells: a literature review. Front Cell Dev Biol. 2021;9:749822. 34966738 10.3389/fcell.2021.749822 PMC8710809

[2] Heng D , Sheng X , Tian C , et al. Mtor inhibition by INK128 extends functions of the ovary reconstituted from germline stem cells in aging and premature aging mice. Aging Cell. 2021;20(2):e13304. 33448083 10.1111/acel.13304 PMC7884035

[3] Zhou L , Xie Y , Li S , et al. Rapamycin prevents cyclophosphamide‐induced over‐activation of primordial follicle pool through PI3K/Akt/mTOR signaling pathway in vivo. J Ovarian Res. 2017;10(1):56. 28814333 10.1186/s13048-017-0350-3 PMC5559863

[4] Huang B , Ding C , Zou Q , Wang W , Li H . Cyclophosphamide regulates N6‐methyladenosine and m6A RNA enzyme levels in human granulosa cells and in ovaries of a premature ovarian aging mouse model. Front Endocrinol. 2019;10:415. 10.3389/fendo.2019.00415 PMC6610338 31316467

[5] Kalich‐Philosoph L , Roness H , Carmely A , et al. Cyclophosphamide triggers follicle activation and "burnout"; AS101 prevents follicle loss and preserves fertility. Sci Transl Med. 2013;5:185. 10.1126/scitranslmed.3005402 23677591

[6] Lambertini M , Boni L , Michelotti A , et al. Ovarian suppression with triptorelin during adjuvant breast cancer chemotherapy and long‐term ovarian function, pregnancies, and disease‐free survival: a randomized clinical trial. JAMA. 2015;314(24):2632‐2640. 26720025 10.1001/jama.2015.17291

[7] Li P , Zhang J , Li F , et al. Low‐intensity ultrasound enhances the chemosensitivity of hepatocellular carcinoma cells to cisplatin via altering the miR‐34a/c‐met axis. Int J Mol Med. 2019;44(1):135‐144. 31115495 10.3892/ijmm.2019.4205 PMC6559300

[8] Jiang X , Savchenko O , Li Y , et al. A review of low‐intensity pulsed ultrasound for therapeutic applications. IEEE Trans Biomed Eng. 2019;66(10):2704‐2718. 30596564 10.1109/TBME.2018.2889669

[9] Chen J , Wang W , Li C , et al. Potential application of low‐intensity pulsed ultrasound in delaying aging for mice. Gerontology. 2022;68(5):558‐570. 34942628 10.1159/000520960

[10] Xu H , Xia Y , Qin J , Xu J , Li C , Wang Y . Effects of low intensity pulsed ultrasound on expression of B‐cell lymphoma‐2 and BCL2‐associated X in premature ovarian failure mice induced by 4‐vinylcyclohexene diepoxide. Reprod Biol Endocrinol. 2021;19(1):113. 34284777 10.1186/s12958-021-00799-w PMC8290625

[11] Qin J , Chen J , Xu H , et al. Low‐intensity pulsed ultrasound promotes repair of 4‐vinylcyclohexene diepoxide‐induced premature ovarian insufficiency in SD rats. J Gerontol A Biol Sci Med Sci. 2022;77(2):221‐227. 34417809 10.1093/gerona/glab242

[12] Zhou W , Chen A , Ye Y , et al. LIPUS combined with TFSC alleviates premature ovarian failure by promoting autophagy and inhibiting apoptosis. Gynecol Endocrinol. 2023;39(1):2258422. 37855244 10.1080/09513590.2023.2258422

[13] Fernandes RD , Hall A , Ferguson M , Lorenzen‐Schmidt I , Balasubramaniam V , Pyle WG . Cardiac changes during the peri‐menopausal period in a VCD‐induced murine model of ovarian failure. Acta Physiol (Oxf). 2019;227(1):e13290. 31050200 10.1111/apha.13290 PMC7379283

[14] Furat Rencber S , Kurnaz Ozbek S , Eraldemır C , et al. Effect of resveratrol and metformin on ovarian reserve and ultrastructure in PCOS: an experimental study. J Ovarian Res. 2018;11(1):55. 29958542 10.1186/s13048-018-0427-7 PMC6025739

[15] Myers M , Britt KL , Wreford NG , et al. Methods for quantifying follicular numbers within the mouse ovary. Reproduction. 2004;127(5):569‐580. 15129012 10.1530/rep.1.00095

[16] Ruth KS , Soares ALG , Borges MC , et al. Genome‐wide association study of anti‐Müllerian hormone levels in pre‐menopausal women of late reproductive age and relationship with genetic determinants of reproductive lifespan. Hum Mol Genet. 2019;28(8):1392‐1401. 30649302 10.1093/hmg/ddz015 PMC6452199

[17] Pan XH , Zhang XJ , Yao X , et al. Effects and mechanisms of mUCMSCs on ovarian structure and function in naturally ageing C57 mice. J Ovarian Res. 2021;14(1):133. 34645513 10.1186/s13048-021-00854-5 PMC8515706

[18] Sullivan SD , Sarrel PM , Nelson LM . Hormone replacement therapy in young women with primary ovarian insufficiency and early menopause. Fertil Steril. 2016;106(7):1588‐1599. 27912889 10.1016/j.fertnstert.2016.09.046 PMC5137796

[19] Wang W , Luo D , Chen J , et al. Amelioration of cyclophosphamide‐induced myelosuppression during treatment to rats with breast cancer through low‐intensity pulsed ultrasound. Biosci Rep. 2020;40(9):BSR20201350. 32936241 10.1042/BSR20201350 PMC7517537

[20] Carolino ROG , Barros PT , Kalil B , Anselmo‐Franci J . Endocrine profile of the VCD‐induced perimenopausal model rat. PLoS One. 2019;14(12):e0226874. 31887176 10.1371/journal.pone.0226874 PMC6936812

[21] Li M , Xie L , Li Y , Liu J , Nie G , Yang H . Synergistic effect of huyang yangkun formula and embryonic stem cells on 4‐vinylcyclohexene diepoxide induced premature ovarian insufficiency in mice. Chinas Med. 2020;15:83. 10.1186/s13020-020-00362-6 PMC7405416 32774448

[22] Wang X , Zhao BS , Roundtree IA , et al. N(6)‐methyladenosine modulates messenger RNA translation efficiency. Cell. 2015;161(6):1388‐1399. 26046440 10.1016/j.cell.2015.05.014 PMC4825696

[23] Wright LE , Christian PJ , Rivera Z , et al. Comparison of skeletal effects of ovariectomy versus chemically induced ovarian failure in mice. J Bone Miner Res. 2008;23(8):1296‐1303. 18348702 10.1359/jbmr.080309 PMC3276352

[24] Ling L , Feng X , Wei T , et al. Human amnion‐derived mesenchymal stem cell (hAD‐MSC) transplantation improves ovarian function in rats with premature ovarian insufficiency (POI) at least partly through a paracrine mechanism. Stem Cell Res Ther. 2019;10(1):46. 30683144 10.1186/s13287-019-1136-x PMC6347748

[25] Wang D , Wang T , Wang R , et al. Suppression of p66Shc prevents hyperandrogenism‐induced ovarian oxidative stress and fibrosis. J Transl Med. 2020;18(1):84. 32066482 10.1186/s12967-020-02249-4 PMC7027222

[26] Liu M , Qiu Y , Xue Z , et al. Small extracellular vesicles derived from embryonic stem cells restore ovarian function of premature ovarian failure through PI3K/AKT signaling pathway. Stem Cell Res Ther. 2020;11(1):3. 31900201 10.1186/s13287-019-1508-2 PMC6942273

[27] Liu T , Han Y , Zhou T , et al. Mechanisms of ROS‐induced mitochondria‐dependent apoptosis underlying liquid storage of goat spermatozoa. Aging. 2019;11(18):7880‐7898. 31548434 10.18632/aging.102295 PMC6782006

[28] Liu SB , Lin XP , Xu Y , Shen ZF , Pan WW . DAXX promotes ovarian cancer ascites cell proliferation and migration by activating the ERK signaling pathway. J Ovarian Res. 2018;11(1):90. 30336783 10.1186/s13048-018-0462-4 PMC6193355

[29] Chen C , Sun X , Xie W , et al. Opposing biological functions of the cytoplasm and nucleus DAXX modified by SUMO‐2/3 in gastric cancer. Cell Death Dis. 2020;11(7):514. 32641734 10.1038/s41419-020-2718-3 PMC7343808

[30] Yang X , Khosravi‐Far R , Chang HY , Baltimore D . Daxx, a novel Fas‐binding protein that activates JNK and apoptosis. Cell. 1997;89(7):1067‐1076. 9215629 10.1016/s0092-8674(00)80294-9 PMC2989411

[31] Liu Z , Wang H , Hu C , et al. Targeting autophagy enhances atezolizumab‐induced mitochondria‐related apoptosis in osteosarcoma. Cell Death Dis. 2021;12(2):164. 33558476 10.1038/s41419-021-03449-6 PMC7870858

[32] Klionsky DJ , Abdel‐Aziz AK , Abdelfatah S , et al. Guidelines for the use and interpretation of assays for monitoring autophagy. Autophagy. 2021;17(1):1‐382. 33634751 10.1080/15548627.2020.1797280 PMC7996087

[33] Kasapoğlu I , Seli E . Mitochondrial dysfunction and ovarian aging. Endocrinology. 2020;161(2):bqaa001. 31927571 10.1210/endocr/bqaa001

[34] Blumenfeld Z , Zur H , Dann EJ . Gonadotropin‐releasing hormone agonist cotreatment during chemotherapy may increase pregnancy rate in survivors. Oncologist. 2015;20(11):1283‐1289. 26463871 10.1634/theoncologist.2015-0223 PMC4718431

[35] Elks CM , Terrebonne JD , Ingram DK , Stephens JM . Blueberries improve glucose tolerance without altering body composition in obese postmenopausal mice. Obesity (Silver Spring). 2015;23(3):573‐580. 25611327 10.1002/oby.20926 PMC4340720

