# Supplementary material for: The potential of low‐intensity pulsed ultrasound to apply the long‐term ovary protection from injury induced by 4‐vinylcyclohexene diepoxide through inhibiting granulosa cell apoptosis
Source: Bioeng Transl Med. 2024 Dec 17;10(3):e10744. doi: 10.1002/btm2.10744 (PMC12079353; doi:10.1002/btm2.10744)
Supplement: Supplementary file 1 — Data S1. [file BTM2-10-e10744-s001.docx]

**Supplementary Material**

**The potential of** **low-intensity pulsed** **ultrasound to apply the long-term** **ovary protection from injury induced by VCD through inhibiting GCs apoptosis**

Juan Deng^1,2^, Juan Qin^3^, Guolin Song^4^, Chenghai Li ^1,2^, Wentao Tang^1,2^, Yilin Tang^1,2^, Xinfang Xiao^1,2^, Liu Wu^1,2^, Sicheng He^1,2^, Yiqing Zhou^1,2^, Junfen Li^1,2^ and Yan Wang^1,2^*

^1^State Key Laboratory of Ultrasound in Medicine and Engineering, Chongqing Medical University, Chongqing, China.

^2^Chongqing Key Laboratory of Biomedical Engineering, Chongqing Medical University, Chongqing, 400016, China.

^3^ Department of Obstetrics and Gynecology, Guiyang Maternal and Child Health Care Hospital, Guizhou Medical University, Guizhou, 550003, China.

^4^ Department of Emergency, The Second Affiliated Hospital of Guizhou University of Traditional Chinese Medicine, Guizhou 550003, China.

*Corresponding author: Yan Wang, State Key Laboratory of Ultrasound in Medicine and Engineering, Chongqing Medical University, 1 Yixueyuan Rd, Yuzhong District, Chongqing, China.

Tel: +86 13883274124

Email: wangyancq@cqmu.edu.cn

**Supplementary Tables**

**Table S1.** Primer sequences of qRT-PCR

| Gene | Primer sequence |
| --- | --- |
| Fas | F: AAGCTCCTTTGGCTGCTGAT  R: TCATAGGTGGCAGGCTCTCT |
| Daxx | F: TGAGGGTCCTAGTGGAGTGG  R: CAAGATGGTGGGGAGGGAAC |
| ASK1 | F: AGGAAGGAGGAGACCGAGTC |
| JNK  Bax  Bcl-2  Cytc  Apaf-1  Caspase 3  Caspase 9  PARP  TBP | R: CATCTCTACCTGCCGAGCTG  F: CCTTCTTGGACGACAGCCAT  R: TGACTGAGTTCGTGTGACCG  F: CCAGGACGCATCCACCAAGAAG  R: GCCACACGGAAGAAGACCTCTC  F: TCCTTCCAGCCTGAGAGCAACC  R: CGACGGTAGCGACGAGAGAAGT  F: TCTGTTTGGGCGGAAGACAG  R: AGCACGGGTGAGTCTTCTTG  F: ATGGGCTCCCTCTCATCAGT  R: TGACTGAGTTCGTGTGACCG  F: AGAACTGGACTGTGGCATTGAGAC  R: GGAATAGTAACCTGGTGCTGTGGAA  F: CACGTGGACTGTGAGAAGCT  R: CCAGGAACCGCTCTTCTTGT  F: TACCATCTGGAGAGTCCGCA  R: CCTCACACACGACTCGAACA  F: CTCAGTTACAGGTGGCAGCA  R: ACCAACAATCACCAACAGCA |

**Supplementary Figures**


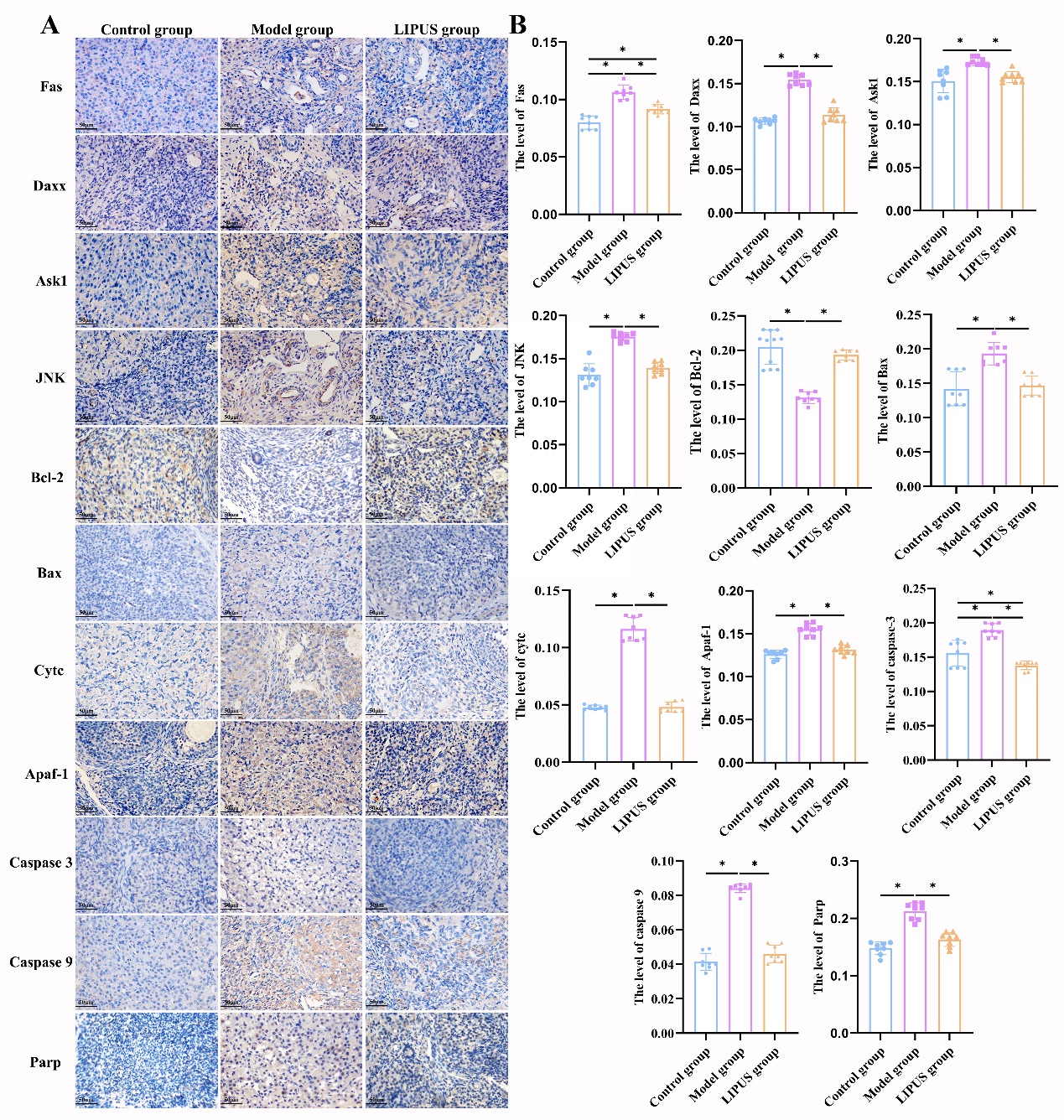


**Fig S1**. LIPUS significantly regulated the expression of Daxx-mediated ASK1/JNK signaling pathway related proteins. (A) Representative immunohistochemical images of proteins involved in the Daxx-mediated ASK1/JNK signaling pathway (400×). (B) Immunohistochemical quantitative analysis. **p* < 0.05.


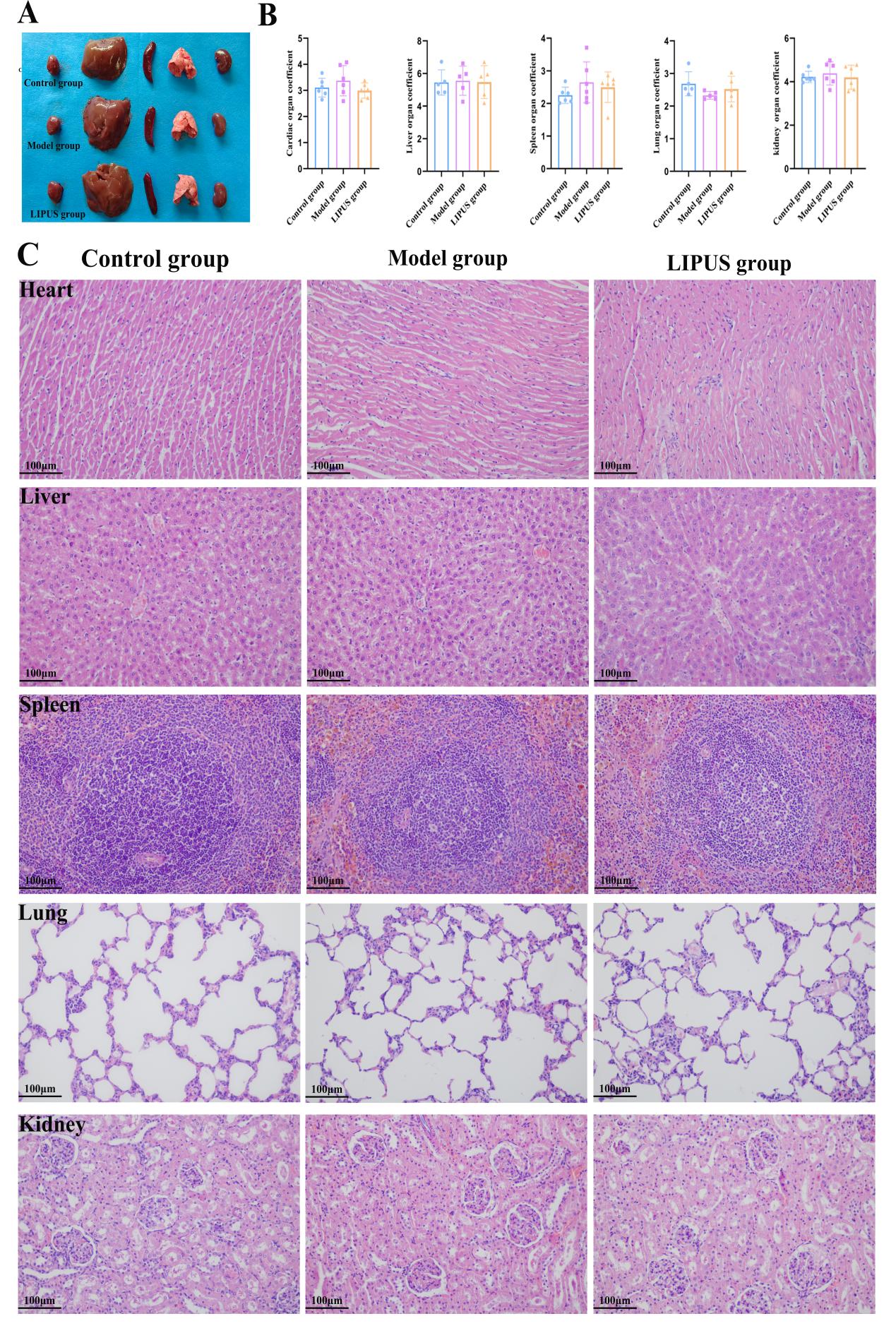


**Fig S2**. LIPUS had no adverse effects on the peripheral organs of rats with VCD-induced ovarian injury. (A) Morphological images of heart, liver, spleen, lung and kidney in each group after LIPUS treatment. (B) Mass coefficient of heart, liver, spleen, lung and kidney in each group after LIPUS treatment. (C) Representative images of H&E staining of heart, liver, spleen, lung and kidney in each group after LIPUS treatment (200×). **p* < 0.05.


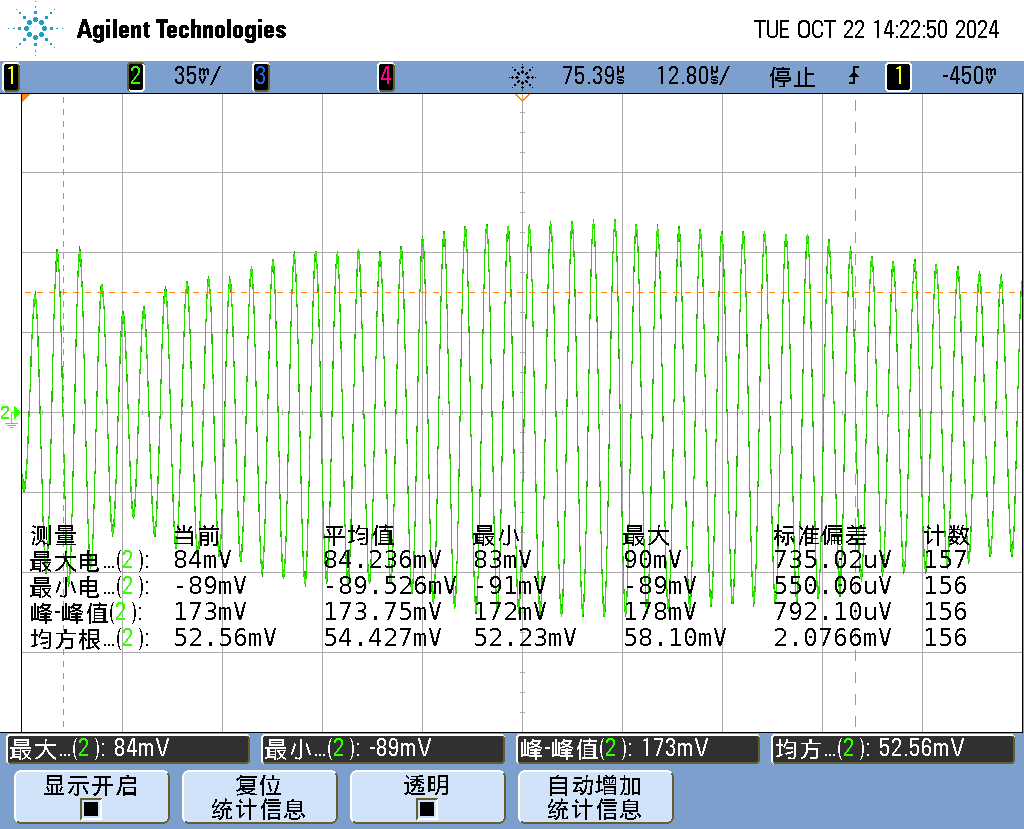


**Fig S3**. The hydrophone measures the focal acoustic pressure map with an acoustic intensity of 200mW/cm^2^.


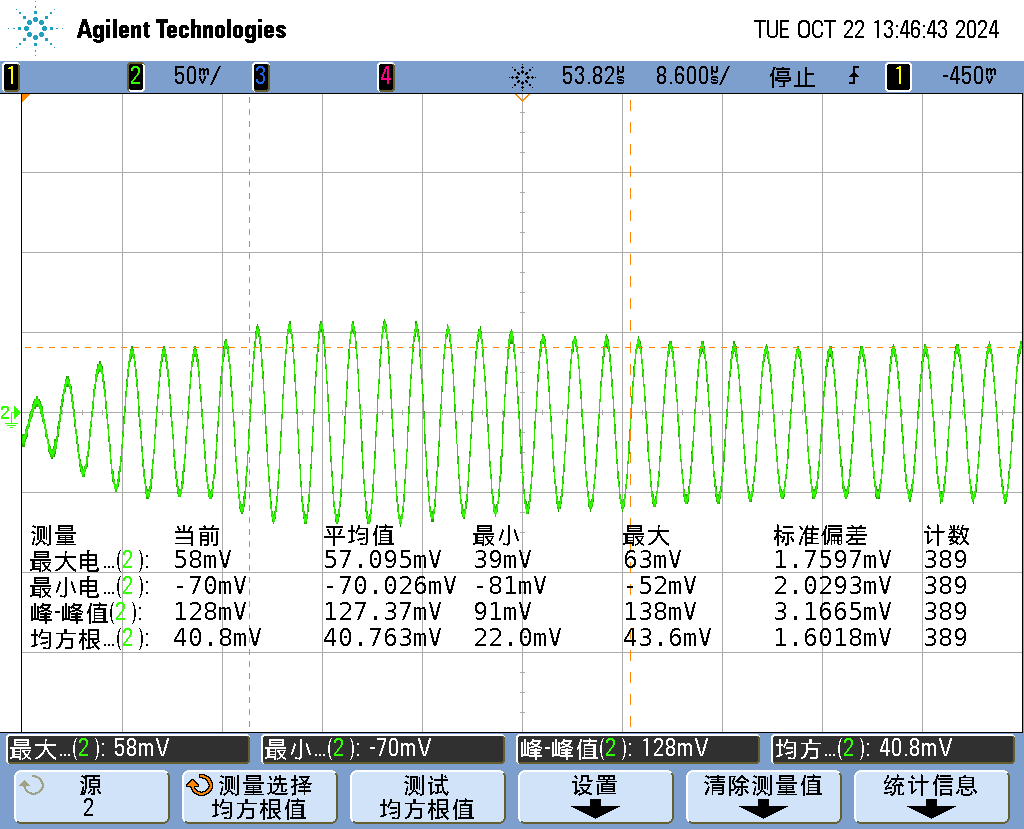


**Fig S4**. The hydrophone measures the focal acoustic pressure map with an acoustic intensity of 30mW/cm^2^.


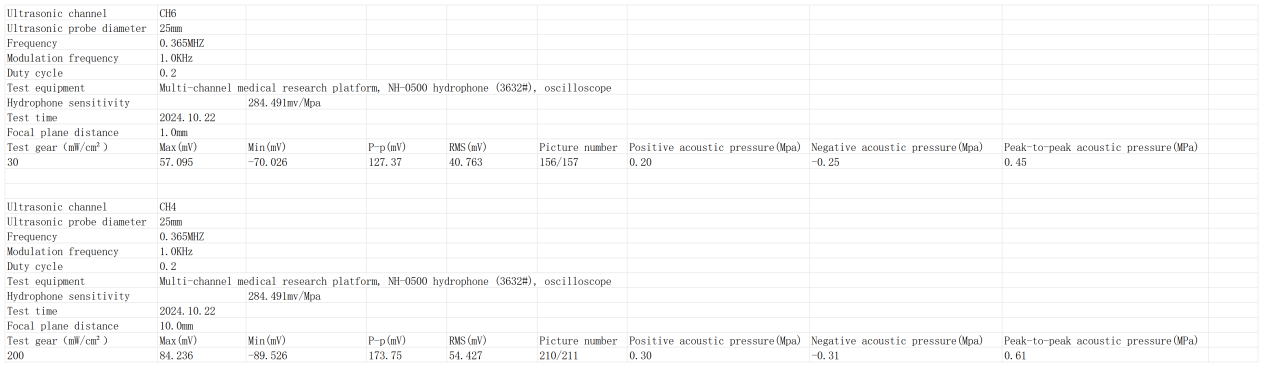


**Fig S5**. The hydrophone measures the focal acoustic pressure data with an acoustic intensity of 30mW/cm^2^ and 200mW/cm^2^ .


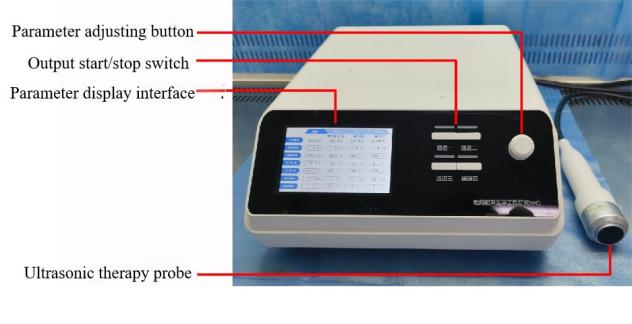


**Fig S6**. Four channel low intensity pulse ultrasonic instrument for animal experiments.


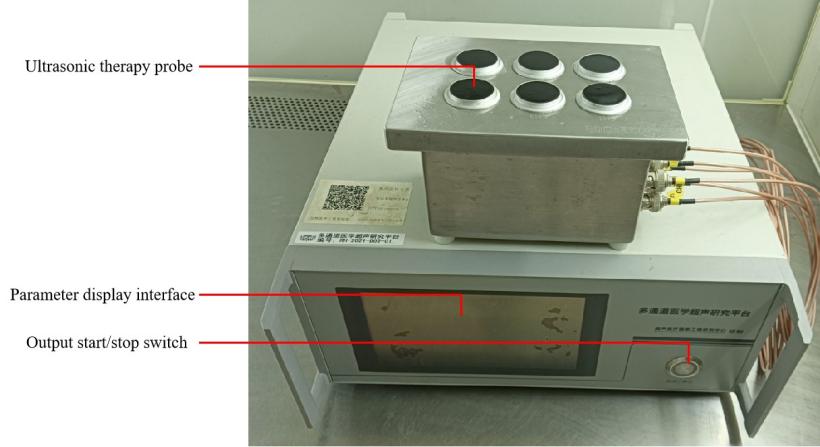


**Fig S7**. Six channel low intensity pulse ultrasonic instrument for cell experiments.

**β-actin（42 kDa）**

**15kDa**

**20kDa**

**25kDa**

**35kDa**

**40kDa**

**50kDa**

**70kDa**

**100kDa**

**150kDa**


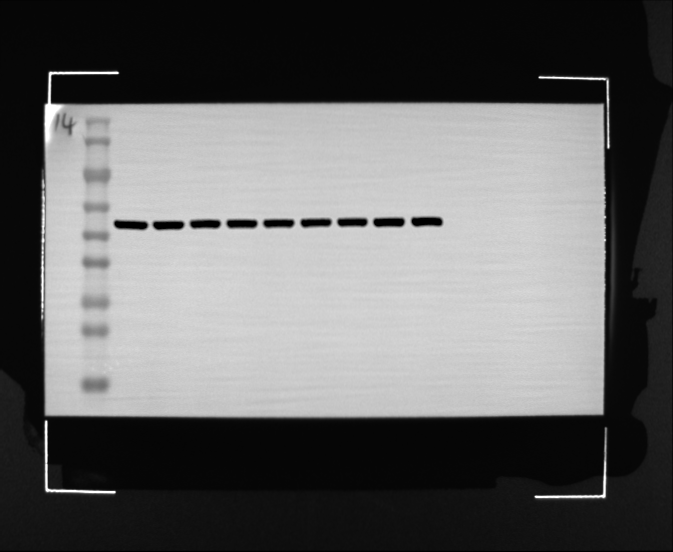


Control group

Model group

LIPUS group

**Apaf-1（120 kDa）**

**15kDa**

**20kDa**

**25kDa**

**35kDa**

**40kDa**

**50kDa**

**70kDa**

**100kDa**

**150kDa**

**
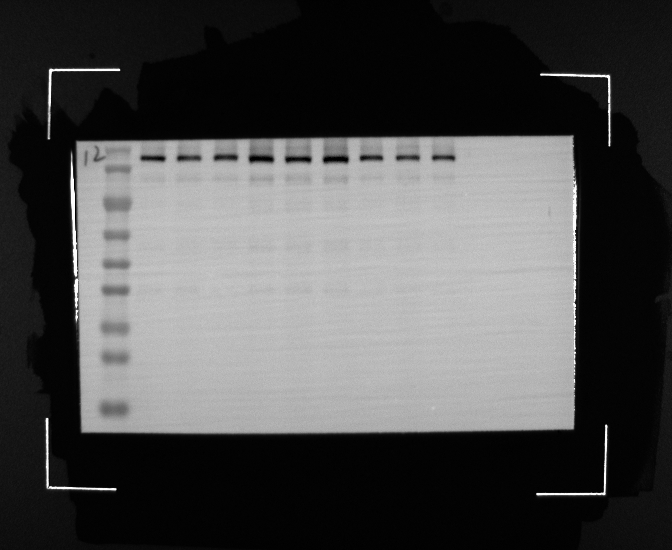
**

Control group

Model group

LIPUS group

**
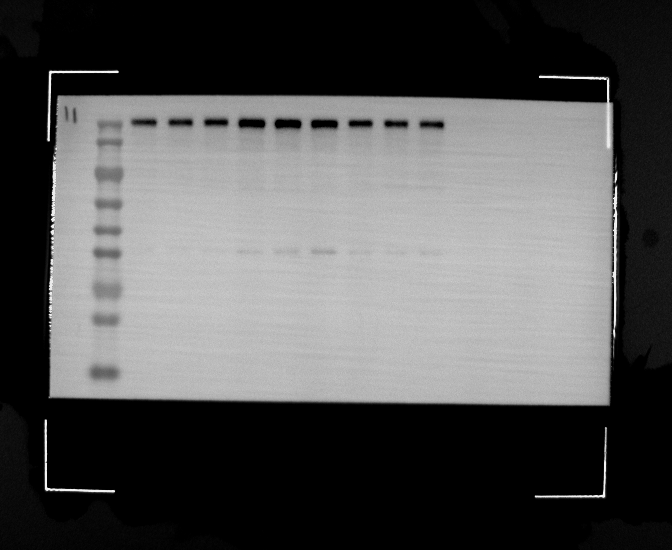
ASK 1（155 kDa）**

**15kDa**

**20kDa**

**25kDa**

**35kDa**

**40kDa**

**50kDa**

**70kDa**

**100kDa**

**150kDa**

Control group

Model group

LIPUS group

**Bax（21 kDa）**

**
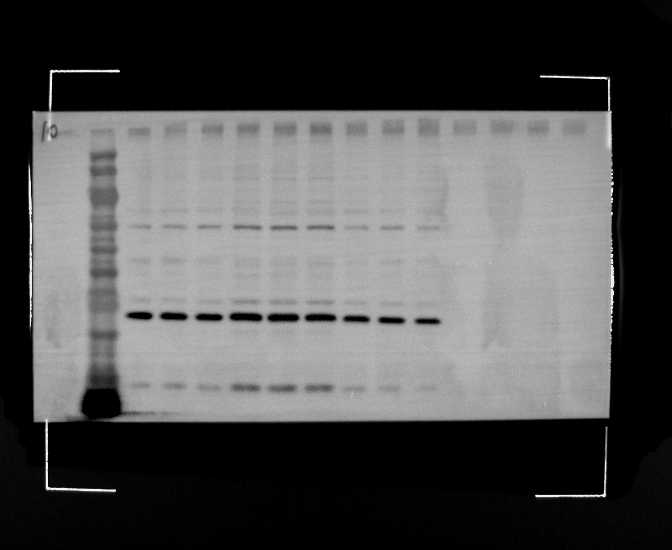
**

**15kDa**

**20kDa**

**25kDa**

**35kDa**

**40kDa**

**50kDa**

**70kDa**

**100kDa**

**150kDa**

Control group

Model group

LIPUS group

**Bcl-2（24 kDa）**

**15kDa**

**20kDa**

**25kDa**

**35kDa**

**40kDa**

**50kDa**

**70kDa**

**100kDa**

**150kDa**

**
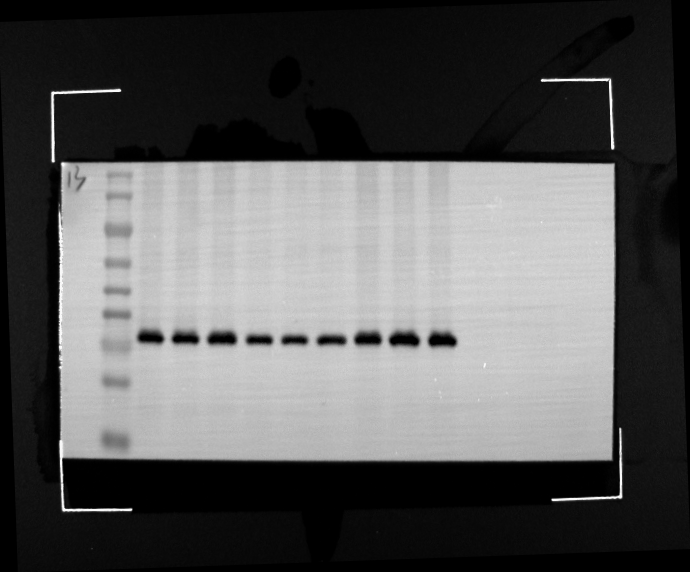
**

Control group

Model group

LIPUS group

**
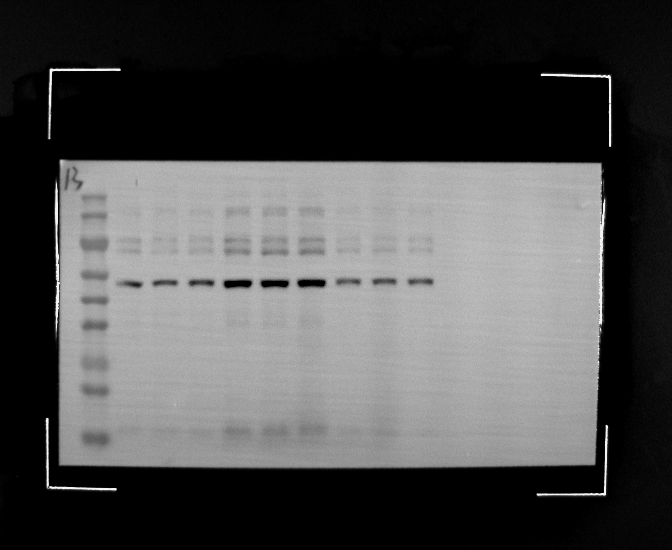
Caspase 9（46 kDa）**

**15kDa**

**20kDa**

**25kDa**

**35kDa**

**40kDa**

**50kDa**

**70kDa**

**100kDa**

**150kDa**

Control group

Model group

LIPUS group

**Cytc（12 kDa）**

**
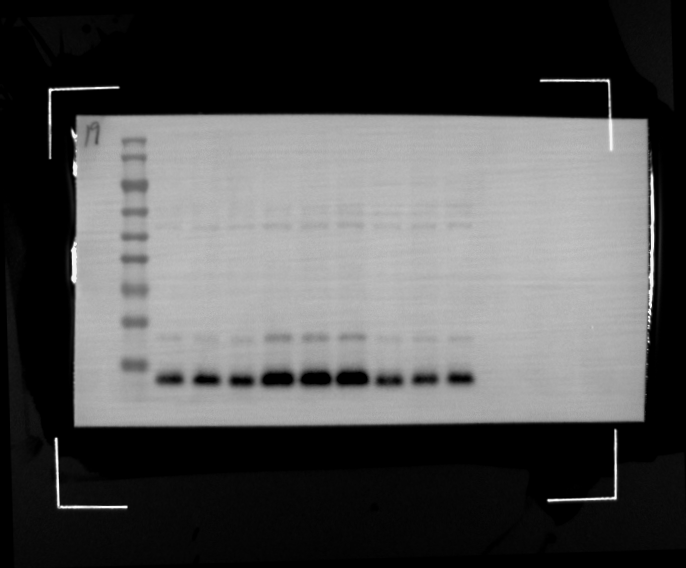
**

**15kDa**

**20kDa**

**25kDa**

**35kDa**

**40kDa**

**50kDa**

**70kDa**

**100kDa**

**150kDa**

**15kDa**

**20kDa**

**25kDa**

**35kDa**

**40kDa**

**50kDa**

**70kDa**

**100kDa**

**150kDa**

Control group

Model group

LIPUS group

**Daxx（82 kDa）**

**
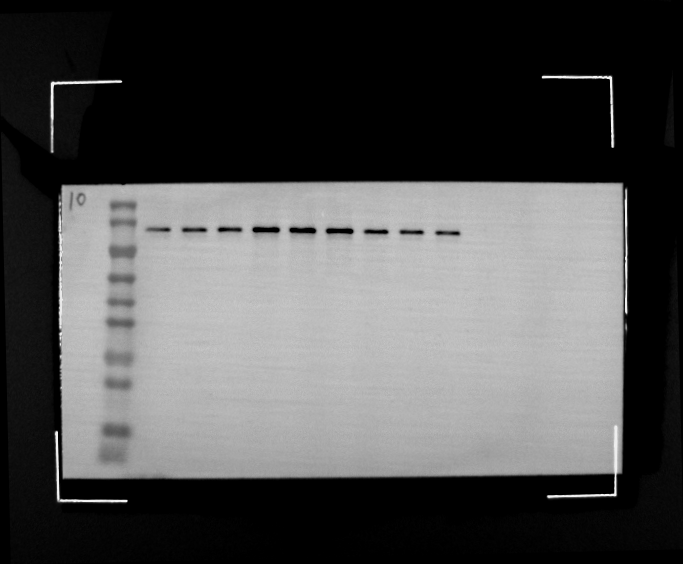
**

**15kDa**

**20kDa**

**25kDa**

**35kDa**

**40kDa**

**50kDa**

**70kDa**

**100kDa**

**150kDa**

Control group

Model group

LIPUS group

**
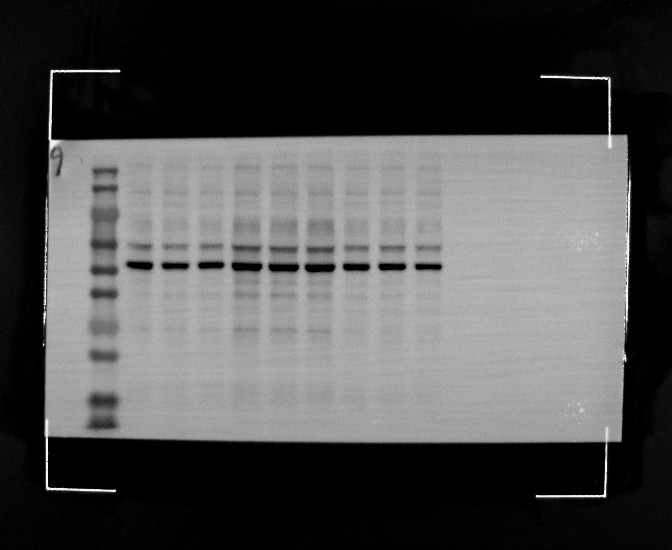
JNK（48 kDa）**

Control group

Model group

LIPUS group

**Parp（113 kDa）**

**15kDa**

**20kDa**

**25kDa**

**35kDa**

**40kDa**

**50kDa**

**70kDa**

**100kDa**

**150kDa**

**
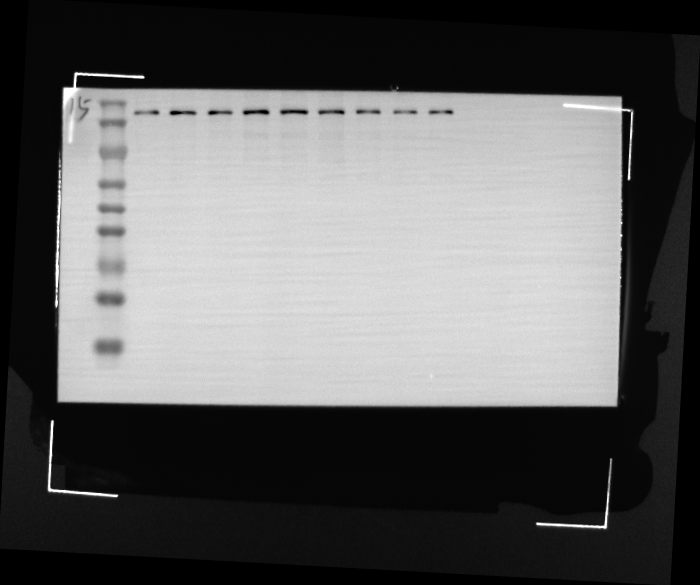
**

Control group

Model group

LIPUS group

**Caspase 3（37 kDa）**


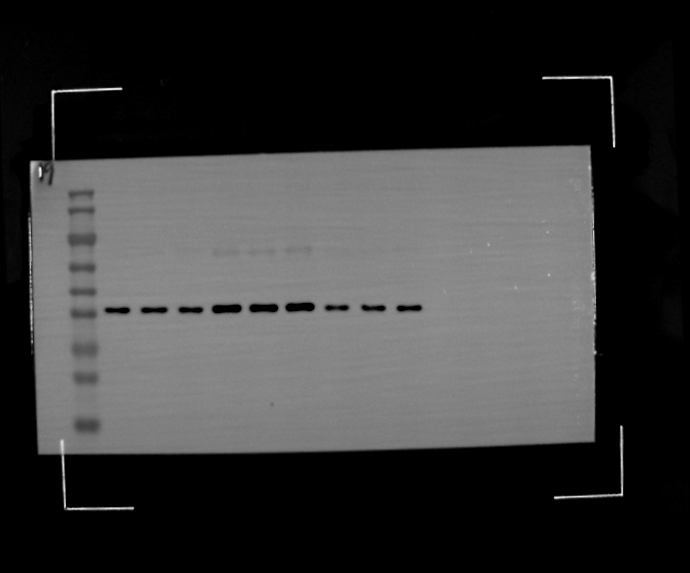


**15kDa**

**20kDa**

**25kDa**

**35kDa**

**40kDa**

**50kDa**

**70kDa**

**100kDa**

**150kDa**

Control group

Model group

LIPUS group

**Fas（38 kDa）**


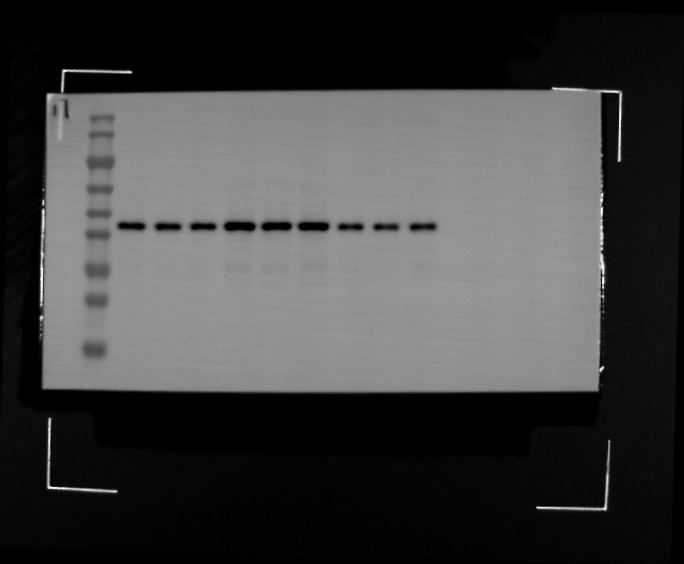


**15kDa**

**20kDa**

**25kDa**

**35kDa**

**40kDa**

**50kDa**

**70kDa**

**100kDa**

**150kDa**

Control group

Model group

LIPUS group

**β-actin（42 kDa）**

Control group

Model group

LIPUS group

Ac-DEVD-CHO group

**
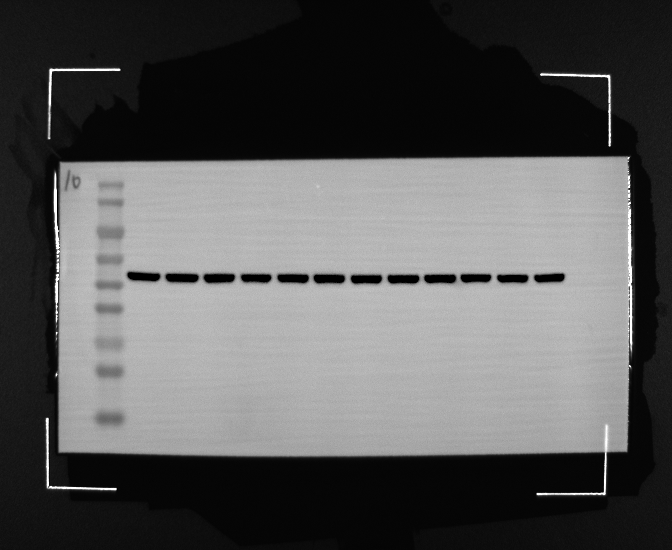
**

**15kDa**

**20kDa**

**25kDa**

**35kDa**

**40kDa**

**50kDa**

**70kDa**

**100kDa**

**150kDa**

**
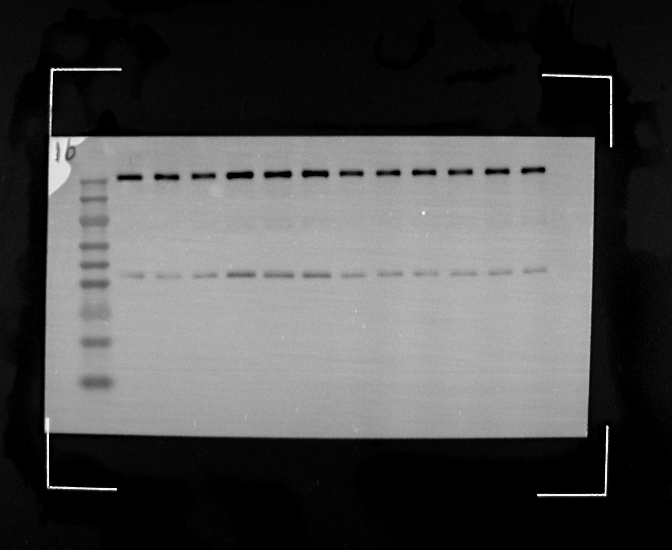
ASK（155 kDa）**

Control group

Model group

LIPUS group

Ac-DEVD-CHO group

**
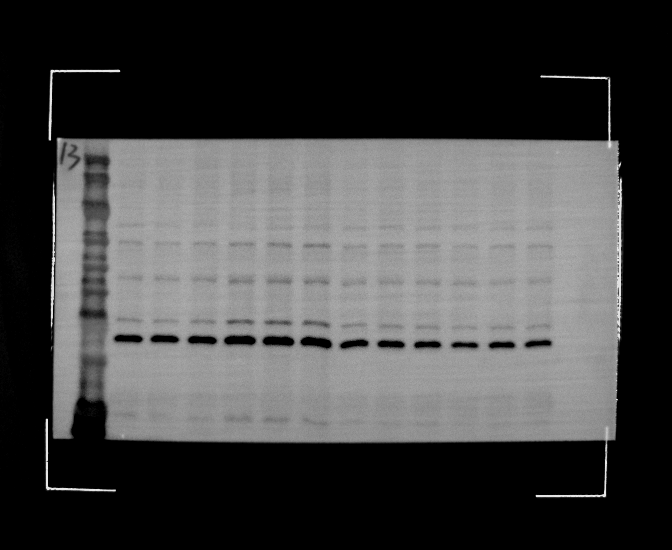
Bax（21 kDa）**

**15kDa**

**20kDa**

**25kDa**

**35kDa**

**40kDa**

**50kDa**

**70kDa**

**100kDa**

**150kDa**

**15kDa**

**20kDa**

**25kDa**

**35kDa**

**40kDa**

**50kDa**

**70kDa**

**100kDa**

**150kDa**

Control group

Model group

LIPUS group

Ac-DEVD-CHO group

**Cytc（12 kDa）**

**
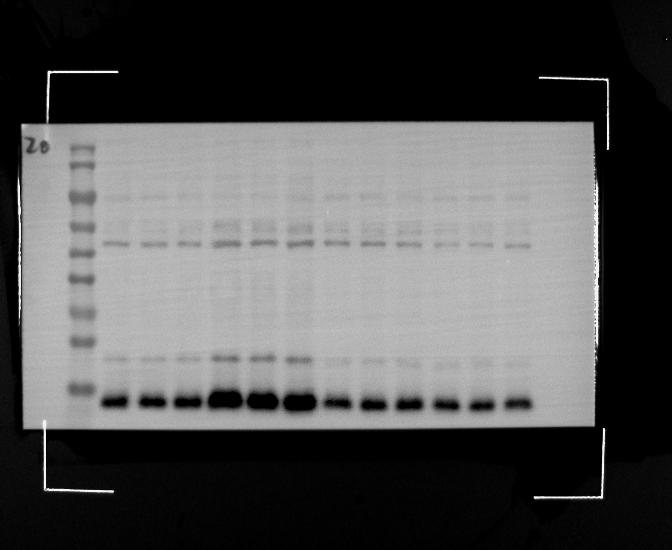
**

**15kDa**

**20kDa**

**25kDa**

**35kDa**

**40kDa**

**50kDa**

**70kDa**

**100kDa**

**150kDa**

Control group

Model group

LIPUS group

Ac-DEVD-CHO group

**Caspase 3（37 kDa）**

**
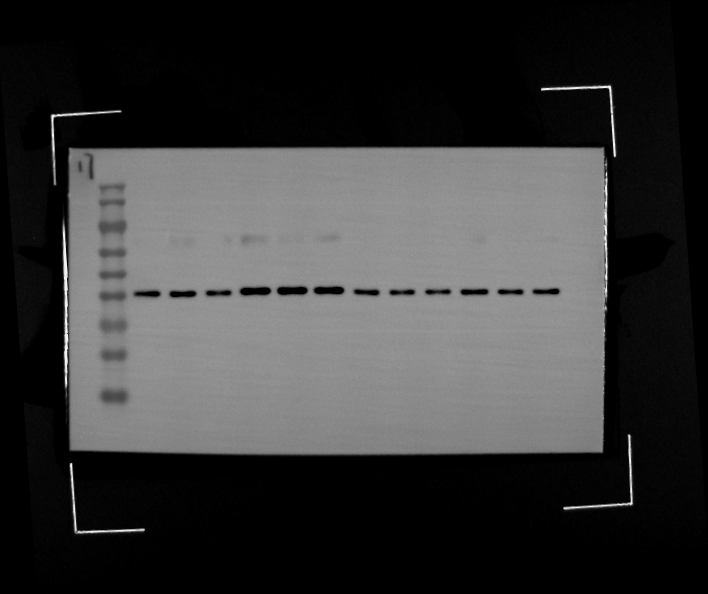
**

**15kDa**

**20kDa**

**25kDa**

**35kDa**

**40kDa**

**50kDa**

**70kDa**

**100kDa**

**150kDa**

Control group

Model group

LIPUS group

Ac-DEVD-CHO group

**
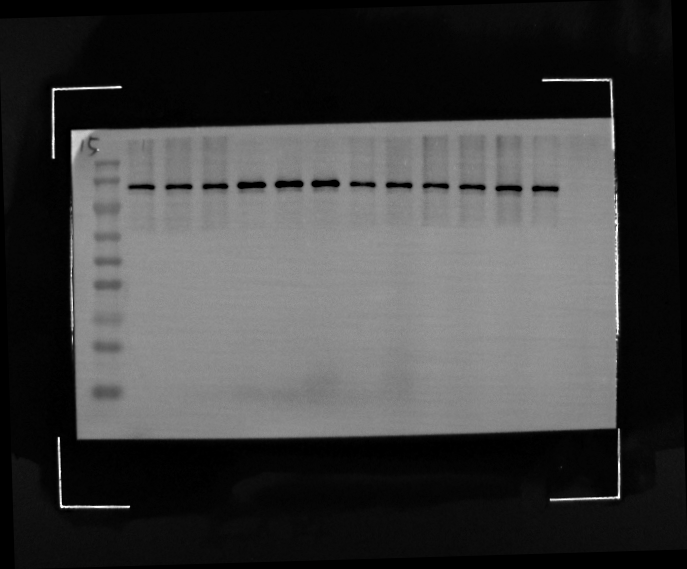
Daxx（82 kDa）**

**15kDa**

**20kDa**

**25kDa**

**35kDa**

**40kDa**

**50kDa**

**70kDa**

**100kDa**

**150kDa**

Control group

Model group

LIPUS group

Ac-DEVD-CHO group

**Fig S8**. The full gel images of β-actin, Apaf-1, ASK 1, Bax, Bcl-2, Caspase 9, Cytc, JNK, JNK, Parp, Caspase 3 and Fas expression in ovarian tissue and cells of each group were detected by WB.
